# Immunogenicity and reactogenicity of intradermal mRNA-1273 SARS-CoV-2 vaccination: a non-inferiority, randomized-controlled trial

**DOI:** 10.1038/s41541-023-00785-w

**Published:** 2024-01-02

**Authors:** Manon L. M. Prins, Geert V. T. Roozen, Cilia. R. Pothast, Wesley Huisman, Rob van Binnendijk, Gerco den Hartog, Vincent P. Kuiper, Corine Prins, Jacqueline J. Janse, Olivia. A. C. Lamers, Jan Pieter R. Koopman, Annelieke C. Kruithof, Ingrid M. C. Kamerling, Romy C. Dijkland, Alicia. C. de Kroon, Shohreh Azimi, Mariet C. W. Feltkamp, Marjan Kuijer, Simon P. Jochems, Mirjam H. M. Heemskerk, Frits R. Rosendaal, Meta Roestenberg, Leo G. Visser, Anna H. E. Roukens

**Affiliations:** 1 Department of Infectious Diseases, Leiden University Medical Center, Leiden, The Netherlands; 2 Department of Parasitology, Leiden University Medical Center, Leiden, The Netherlands; 3 Department of Haematology, Leiden University Medical Center, Leiden, The Netherlands; 4 Department of Immune Surveillance, National Institute for Public Health and the Environment, Bilthoven, The Netherlands; 5 Laboratory of Medical Immunology, RadboudUMC, Nijmegen, The Netherlands; 6 Center for Human Drug Research, Leiden, The Netherlands; 7 Department of Medical Microbiology, Leiden University Medical Center, Leiden, The Netherlands; 8 Department of Clinical Epidemiology, Leiden University Medical Center, Leiden, The Netherlands

**Keywords:** Randomized controlled trials, Viral infection

## Abstract

Fractional dosing can be a cost-effective vaccination strategy to accelerate individual and herd immunity in a pandemic. We assessed the immunogenicity and safety of primary intradermal (ID) vaccination, with a 1/5th dose compared with the standard intramuscular (IM) dose of mRNA-1273 in SARS-CoV-2 naïve persons. We conducted an open-label, non-inferiority, randomized controlled trial in the Netherlands between June and December 2021. One hundred and fifty healthy and SARS-CoV-2 naïve participants, aged 18–30 years, were randomized (1:1:1) to receive either two doses of 20 µg mRNA-1273 ID with a standard needle (SN) or the Bella-mu® needle (BM), or two doses of 100 µg IM, 28 days apart. The primary outcome was non-inferiority in seroconversion rates at day 43 (D43), defined as a neutralizing antibody concentration threshold of 465 IU/mL, the lowest response in the IM group. The non-inferiority margin was set at −15%. Neutralizing antibody concentrations at D43 were 1789 (95% CI: 1488–2150) in the IM and 1263 (951–1676) and 1295 (1020–1645) in the ID-SN and ID-BM groups, respectively. The absolute difference in seroconversion proportion between fractional and standard-dose groups was −13.95% (−24.31 to −3.60) for the ID-SN and −13.04% (−22.78 to −3.31) for the ID-BM group and exceeded the predefined non-inferiority margin. Although ID vaccination with 1/5th dose of mRNA-1273 did not meet the predefined non-inferior criteria, the neutralizing antibody concentrations in these groups are far above the proposed proxy for protection against severe disease (100 IU/mL), justifying this strategy in times of vaccine scarcity to accelerate mass protection against severe disease.

## Introduction

Safe and effective vaccines have proven to be the cornerstone of success in the battle against SARS-CoV-2 during the COVID-19 pandemic, but vaccine inequity remains a challenge across the globe^1,2^. Vaccine dose-sparing techniques, such as intradermal (ID) administration, may offer an important advantage in (emergency) mass immunization campaigns as more people can be vaccinated with the same stockpile, with the potential additional advantage of fewer side effects^3^. Modeling has shown that, even if vaccine efficacy of fractional dose is lower than that of full dose vaccination, fractional dosing of SARS-CoV-2 vaccines could be a very cost-effective vaccination strategy and reduce a large number of deaths in lower- and middle-income countries^4^.

In ID administration, the vaccine is introduced directly into the papillary dermis, where antigen-presenting cells are abundantly present. A 1/10th or 1/5th fractional vaccine dose can induce protective immune responses equivalent to the standard dose delivered intramuscularly (IM), as has been shown for many vaccines such as rabies, yellow fever, poliomyelitis, and seasonal influenza vaccine^5^. Since ID delivery is considered technically more difficult than IM vaccination, novel ID devices are being developed^6^. We chose an mRNA-1273 vaccine for ID delivery because at the beginning of the COVID-19 pandemic, only mRNA vaccines (mRNA-1273 and BNT162b2 vaccine) were available in the Netherlands. In addition, ID delivery had never been studied with an mRNA vaccine and if this was safe and effective, it could have major implications for the future of mRNA vaccines.

Recently, we demonstrated the safety and immunogenicity of two doses of 10 µg or 20 µg mRNA-1273 at 28-days-interval through the ID route in a proof-of-concept study^7^. The SARS-CoV-2-spike-S1 and -RBD IgG-binding antibodies generated by 10 µg or 20 µg mRNA-1273 vaccine ID were similar in magnitude to the levels seen in subjects from an age-matched cohort vaccinated with 100 µg IM. These results justified a larger randomized-controlled, non-inferiority study. We investigated whether virus-neutralizing antibody and binding antibody concentration elicited by two 1/5th doses of mRNA-1273 vaccine given at a 28-day interval by ID vaccination were non-inferior to those of a control group receiving two standard doses of mRNA-1273 vaccine. Additionally, we measured SARS-CoV-2-specific memory B- and T-cell responses. Finally, we evaluated the performance of an easy-to-use ID microneedle to facilitate ID delivery on a wider scale.

## Results

### Trial population

**Fig. 1:**
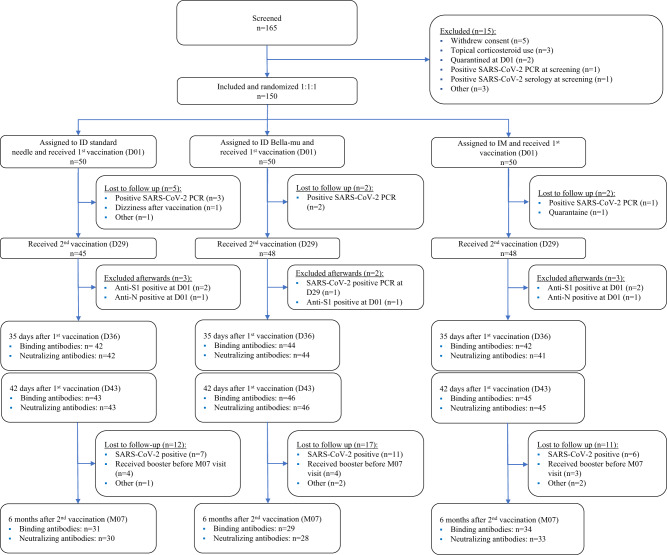
Flowchart of inclusions. Among the 141 participants receiving a second vaccination, 7 participants were excluded from the immunogenicity analysis afterward due to seropositivity for IgG anti-S1 or anti-N at baseline, indicating an earlier unrecognized SARS-CoV-2 infection. One of them was one of the two participants who ended the study prematurely due to dizziness. ID intradermal, IM intramuscular, D day, M months.

Between June 14th and July 8th of 2021, 165 participants were assessed for eligibility (Fig. 1). One-hundred and fifty eligible participants were enrolled and randomized to receive either 20 µg mRNA-1273 ID-SN (*n* = 50), 20 µg mRNA-1273 ID-BM (*n* = 50) or 100 µg mRNA-1273 IM (*n* = 50). The participants' characteristics are shown in Supplementary Table 8. The median age was 22 years, and 63/150 (42%) of participants were female. All 150 participants received at least one vaccine dose. One hundred and forty-one participants (94%) received a second dose and completed all scheduled safety visits.

Several participants were excluded from the analysis for various reasons. This included participants with a positive SARS-CoV-2 PCR before D29 (*n* = 6), participants who showed signs of past SARS-CoV-2 infection based on baseline seropositivity for anti-S1 and/or anti-N IgG antibodies (*n* = 7), and two seronegative participants from the IM group who displayed activated SARS-CoV-2-spike-specific B-cells prior to vaccination, indicating recent infection. These two participants were excluded in the PP in-depth B-cell analysis but not from the immunogenicity analysis to avoid bias, as in-depth B-cell analysis was not performed in all participants. The ITT population for immunogenicity at D43 included 134 participants. Forty participants were excluded between D43 and M07, mainly due to intercurrent SARS-CoV-2 infection (*n* = 24) or booster vaccination through the national vaccination campaign (*n* = 11). The immunogenicity analysis at M07 included 94 participants who were tested for IgG-binding antibodies, 91 of which were also analyzed for virus neutralization.

All injections were considered successful, however, ID vaccination with the Bella-mu® needle elicited slightly smaller wheals (8 mm; IQR: 7–9; 95% CI: 8–9) than standard technique ID vaccination (9 mm; IQR: 9–10; 95% CI: 9–9).

### Neutralization and binding antibody responses

The seroconversion rate at D43 was 100% in the IM group, whereas in the ID-SN and ID-BM groups, it was 86% (95% CI: 73.2–94.1) and 87% (74.8–94.5) (Table 1). The lower limit of the 95% CI for the difference in response compared with the IM group exceeded the predefined non-inferiority margin for both ID groups.

**Table 1:** Seroconversion and neutralization (IU/mL) in fractional and standard doses at day 43.

	Total (*n*)	Seroconversion			Neutralization concentration IU/mL (95% CI)
		*n*	% (95% CI)	Difference in response (%)	
20 µg ID-SN	43	37	86% (73.2–94.1)	−13.95% (24.31 to −3.60)	1263 (951–1676)
20 µg ID-BM	46	40	87% (74.8–94.5)	−13.04% (−22.78 to −3.31)	1295 (1020–1645)
100 µg IM	45	45	100 (93.6–100.0)	Ref.	1789 (1488–2150)

*n* number of participants, *ID* intradermal, *IM* intramuscular, *SN* standard needle, *BM* Bella-mu® needle, *Ref.* reference group, *CI* confidence interval, *IU/mL* international units per mL.

**Fig. 2:**
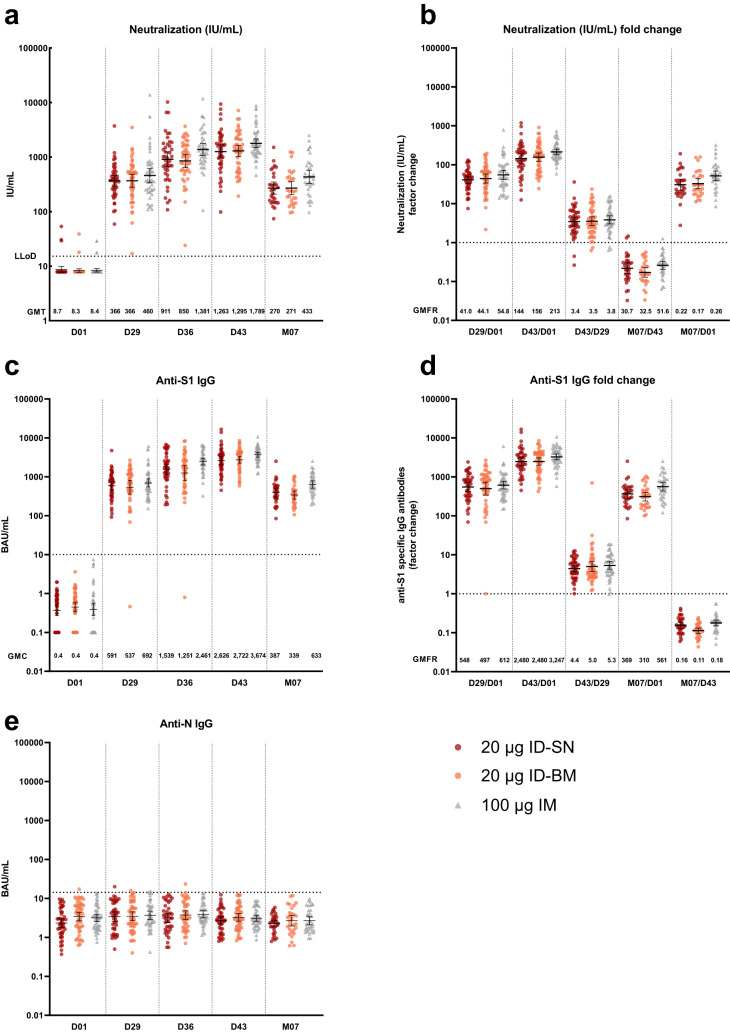
SARS-CoV-2 specific immune responses. **a** Virus neutralization concentration in international units per mL. Horizontal dotted lines represent the LLoD (=15.26 IU/mL). Results below the LLoD were arbitrarily set to LLoD/2. **b** Neutralization concentration fold change. The dashed line indicates a factor change of 1 (no increase or decrease). Horizontal lines represent the geometric mean + 95% CI of the geometric mean. **c** SARS-CoV-2 S1-specific IgG antibody concentrations by bead-based multiplex immunoassay (MIA) in binding antibody units per mL in the three groups at each timepoint. Horizontal dotted lines represent the cut-off for seropositivity (=10.08 BAU/mL). Horizontal lines represent the geometric mean + 95% CI of the geometric mean. **d** Per-participant factor changes for anti-S1-specific binding antibodies, calculated by dividing two responses. The dashed line indicates a factor change of 1 (no increase or decrease). Horizontal lines represent the geometric mean + 95% CI of the geometric mean. **e** SARS-CoV-2 anti-N-specific IgG antibody concentrations by bead-based immunoassay (MIA) in binding antibody units per mL in the three groups at each timepoint. Horizontal dotted lines represent the cut-off for seropositivity (=14.3 BAU/mL). Horizontal lines represent the geometric mean + 95% CI of the geometric mean. For the calculations of the GMFR D29/D01, D43/D01, and M07/D01, any antibody concentration for S1 and RBD at D01 reported below 1 was set to 1. For the calculation of the GMFR D43/D29, the antibody concentration for S1 for the non-responder in the ID-BM group at D29 was set to 1. Each symbol represents a sample from an individual participant. Black symbols in the IM group represent the two participants with SARS-CoV-2-spike-specific B-cells at baseline but no measurable anti-S or anti-N. ID intradermal, IM intramuscular, BM Bella-mu® needle, SN standard needle, LLoD lower limit of detection, IU/mL international units per mL, D day, M months.

GMCs of neutralizing antibodies at D43 were highest in the standard dose IM group (Fig. 2a, Supplementary Table 9), with mean concentrations of 1789 (1488–2150) in the standard dose IM group and 1263 (95% CI 951–1676) and 1295 (1020–1645) in the ID-SN and ID-BM groups, respectively, with overlapping 95% CIs. At D43, GMCs of IgG-binding antibodies against SARS-CoV-2-spike-S1 were lower in the fractional dose ID groups than in the standard dose IM group, but 95% CIs were also overlapping (Fig. 2c, Supplementary Table 9). Similar results were observed for antibodies against SARS-CoV-2-spike-RBD (Supplementary Fig. 4, Supplementary Table 9).

At M07, GMCs remained elevated in all groups and were highest in the IM group (433; 95% CI 328–573) compared to both ID groups: 270 (209–349) in the ID-SN and 271 (205–359) in the ID-BM group, with overlapping 95% CI’s. Similar results were observed for the GMCs of the SARS-CoV-2 binding antibodies.

The change in GMCs between the different timepoints is shown in Fig. 2b and Supplementary Table 10.

### B-cell responses

**Fig. 3:**
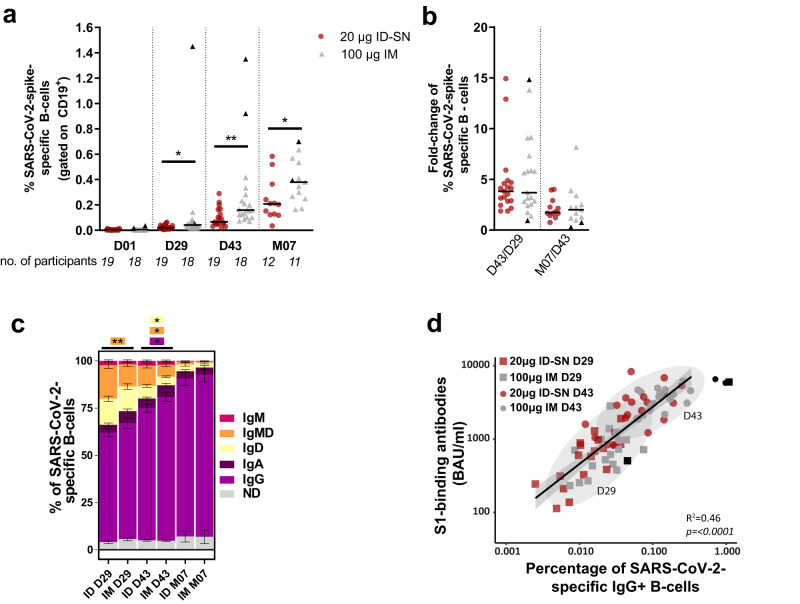
B-cell compartment and the immunogenicity of intradermal and intramuscular delivery of mRNA SARS-CoV-2 vaccine, according to the per-protocol analysis. In total, 40 individuals were selected to investigate the B-cell response against the IM (100 µg) or ID (20 µg) standard delivery technique (ID-SN) of the SARS-CoV-2 vaccine. 12 individuals withdrew from the study and two individuals (IM and ID-SN group) were excluded at 7 months and were not shown due to a recent re-infection (PCR+). Vaccines were administered directly after sample collection at D01 and D29 (booster). The black data points represent the two individuals with the presence of SARS-CoV-2-specific B-cells at D01 prior to vaccination, which were excluded in the PP analysis. Including these participants in the ITT analysis did not change the outcome. **a** Percentages of B-cells specific for SARS-CoV-2-spike-protein, shown as frequencies from total B-cells per individual and vaccine delivery (IM, gray vs ID-SN, red). **b** The fold-change of the frequencies of SARS-CoV-2-spike-specific B-cells, 2 weeks after the second dose (D43/D29) and ~6 months after D43 (M07/D43). **c** Isotype usage of SARS-CoV-2-spike-specific B-cells as stacked bars at each timepoint for each vaccine delivery. **d** Correlation plot between IgG+ titers and IgG+ SARS-CoV-2-spike-specific B-cells. 95% CI is shown as an ellipse for each timepoint. Pearson correlation analysis results are depicted, and linear regression results are shown as a black line with shaded 95% CIs. Statistical analyses and Mann–Whitney *U* tests are performed to compare the vaccine deliveries (ID-SN and IM) for each timepoint. *=*P* < 0.05, **=*P* < 0.01. Individuals and median values are shown. ID intradermal, IM intramuscular, SN standard needle, CI confidence interval, D day, M month, PP per-protocol, ITT intention-to-treat.

Higher frequencies of SARS-CoV-2-spike-specific B-cells were detected at D29, D43, and at M07 in participants receiving IM vaccination, compared to ID-SN vaccinated participants (Fig. 3a). The frequencies of SARS-CoV-2-spike-specific B-cells increased further during the 7 months after first vaccination in both groups and the fold-change of percentages of SARS-CoV-2-spike-specific B-cells at D43/29 and M07/D43 were similar between groups (Fig. 3b). Participants that received ID-SN vaccination had significantly more unswitched SARS-CoV-2 spike-specific B-cells at D29 (IgMD) and D43 (IgD and IgMD), and significantly fewer IgG-switched B-cells at D43, than IM vaccinated participants (Fig. 3c). No significant differences between isotypes were observed at M07, with almost all SARS-CoV-2 spike-specific B-cells switched to IgG in both groups. Percentages of IgG-positive SARS-CoV-2-specific B-cells correlated with the anti-S1-specific IgG antibody concentrations (Fig. 3d).

### T-cell responses

**Fig. 4:**
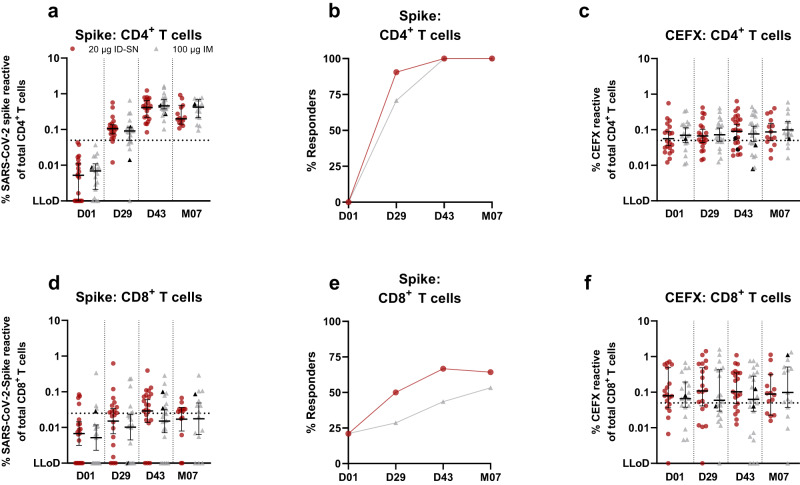
mRNA-1273 induced SARS-CoV-2-specific T-cell responses. Spike-specific CD4^+^ and CD8^+^ T-cells were analyzed at D01, D29, D43 or M07. The second dose was given at the moment of D29. A pool of peptides derived from CMV, EBV, Flu and extra (CEFX) was used as a positive control and DMSO as a negative control. **a** Frequency of spike-specific CD4^+^ T-cells in time. Spike-specific CD4^+^ T-cells were defined as the frequency of CD154^+^ and/or CD137^+^ cells of total CD4^+^ T-cells, corrected for background in DMSO control. The dotted line represents the threshold for a response. **b** Frequency of individuals with a spike-specific CD4^+^ T-cell response above threshold. **c** Frequency of CEFX-specific CD4^+^ T-cells in time. **d** Frequency of spike-specific CD8^+^ T-cells in time. Spike-specific CD8^+^ T-cells were defined as the frequency CD69^+^ and/or CD137^+^ cells of total CD8^+^ T-cells, corrected for background in DMSO control. The dotted line represents the threshold for a response. **e** Frequency of individuals with a spike-specific CD8^+^ T-cell response above threshold. **f** Frequency of CEFX-specific CD8^+^ T-cells in time. Each point represents a single subject. Black symbols in the IM group represent the two participants with suspected previous SARS-CoV-2 infection based on SARS-CoV-2-spike-specific B-cells prior to vaccination. Mann–Whitney *U* tests were performed for statistical analysis. *p*-Values are categorized in the figures as: *p < 0.05; **p < 0.01 or ***p < 0.001. Categorized *p*-value was only shown if significant. The horizontal bold black line represents the median with 95% CI. The dotted line indicates the limit of quantification (LOQ). ID intradermal, IM intramuscular, D day, M months, SN standard needle, LLoD lower limit of detection, CI confidence interval.

Frequencies of spike-specific CD4^+^ T-cells increased with each dose in both groups until D43 and decreased slightly at M07 (Fig. 4a). At D43 and M07, all IM and ID-SN vaccinated individuals had a SARS-CoV-2-spike-specific CD4^+^ T-cell response above threshold (Fig. 4b).

In general, the SARS-CoV-2-specific CD8^+^ T-cell responses were lower and more variable compared to CD4^+^ T-cell responses (Fig. 4d, e and Supplementary Fig. 10C). For more details on the in-depth analysis of the B- and T-cell response, see the Supplementary Appendix (Supplement I and J).

### Vaccine safety

**Fig. 5:**
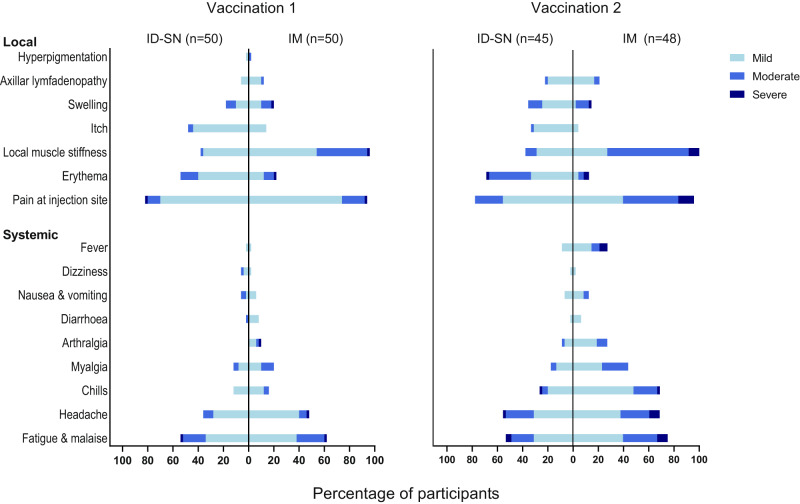
Adverse events related to vaccine administration in the ID-SN group and IM group, subdivided into mild, moderate, or severe. All adverse events possibly, probably, or definitely related to the vaccination in the following 28 days after the first and second vaccine administration are reported. Grade 4 (potentially life-threatening) adverse events did not occur. Hyperpigmentation, itch, and dizziness are unsolicited adverse events. For a number of adverse events in the ID-BM group, *see* Supplementary Table 13. ID intradermal, IM intramuscular, BM Bella-mu® needle, SN standard needle.

No serious AEs or severe COVID-19 cases were reported, and no pre-specified stopping rules were met. Solicited local and systemic AEs were mostly mild or moderate and transient in nature both after the first and second vaccination (Fig. 5 and Supplementary Table 13). Twenty-three of 150 participants (15.3%) had one or more severe (grade 3) AEs (Supplementary Tables 15 and 16), which were self-limiting and resolved within a few days.

Frequencies of AEs in the ID-SN and ID-BM groups were more or less the same (Supplementary Table 13). The three most commonly reported local AEs after ID injection were pain, erythema, and itch at the injection site (Fig. 5). Systemic AEs such as fatigue and malaise, headache, and chills, were more frequently reported in the IM group, especially after the second vaccination. The most common systemic solicited AEs after ID vaccination were fatigue and headache.

## Discussion

Intradermal delivery of two 1/5th fractional doses of the mRNA-1273 vaccine given at a 28-day interval, either by standard needle or Bella-mu® 1.4 microneedle, elicited high levels of neutralizing antibody concentrations at D43 but did not meet non-inferior criteria compared with two standard doses of mRNA-1273 IM. In addition, SARS-CoV-2 B-cells were also slightly lower in the ID groups, but T-cell responses were comparable. Finally, ID vaccination elicited milder systemic AEs.

To our knowledge, this is the first randomized-controlled study in which the immunogenicity reactogenicity and in-depth T- and B-cell responses were evaluated after a primary ID vaccination series with a fractional dose of mRNA-1273 vaccine. A study from Thailand evaluating different homo- and heterologous IM and ID regimens as primary series demonstrated that two ID doses generated similar SARS-CoV-2 anti-RBD IgG-antibodies as their respective standard IM-IM regimens, except for homologous BNT162b2 delivered ID^8^. However, the mRNA-1273 vaccine regimen was not evaluated.

Both binding and neutralizing antibodies have been proposed as a proxy for protection (CoP) against symptomatic and severe COVID-19 disease^9–13^. However, all studies found that the level of protection evolved gradually with neutralization titer. Consequently, no specific cut-off level exists below which individuals lack protection or above which protection is guaranteed. In addition, establishing a universal threshold in international units poses challenges due to the absence of standardized assays across various studies^14^. At the start of this study, no cut-off level regarding neutralizing antibodies was known. Therefore, the definition of seroconversion for this study was based on a study from Jackson et al.^15^ using the plaque reduction test, which is different from the MNA used in our study. Since the predefined seroconversion could not be used in our assessment of non-inferiority, we chose the lowest neutralization concentration of the IM group (control group) as the cut-off for seroconversion, which was 465 IU/mL. Analysis of the phase 3 study of mRNA-1273 suggested that protection against symptomatic COVID-19 disease was 91% and 96% with a day 57 neutralizing antibody concentration of 100 and 1000 IU/mL (50% virus neutralization), respectively^11,16^. In addition, Gilbert et al. estimated that a level of 300 BAU/mL at day 57 was associated with 90% protection against symptomatic COVID-19 (D614G variant) by the mRNA-1273 vaccine^16^. In our study (during the wave with the Delta variant), all participants developed an adequate SARS-CoV-2-spike-S1 binding antibody concentration above 300 BAU/mL, and all except one participant (of the ID-SN group) showed a neutralizing antibody concentration above 100 IU/mL at D43, indicating a high level of protection in all groups, despite not meeting the pre-defined non-inferiority criteria.

Cellular immunity plays a key role in controlling disease severity. Thus, analyzing B- and T-cell responses is necessary to provide further insight into the effectiveness and durability of the adaptive immune response^17^. Evidence also indicates that T-cell responses are less likely to be affected by spike antigen mutations associated with variants of concern (VOC) compared to antibody response^17–19^. We showed that priming with the first vaccine dose resulted in a lower frequency of SARS-CoV-2-specific B-cells in both ID groups at all follow-up time points. However, the response was equally effective and the immunization kinetics were comparable, with similar phenotypical SARS-CoV-2-specific B-cells to standard dose IM delivery. Both the 20 µg and the 100 µg dose elicited a rapid CD4^+^ response after the first and second vaccination, consistent with other studies^12,15,18,20–24^.

Also consistent with other studies^7,8^, we observed more local AEs with ID than IM vaccination; however, these were predominantly mild or moderate. More importantly, ID administration led to a lower incidence of systemic AEs than IM vaccination. This could have important consequences, as fewer systemic side effects may lead to less absenteeism and higher vaccine acceptance in vaccine-hesitant individuals^25,26^.

The Bella-Mu® microneedle showed comparable results regarding immunogenicity and safety when comparing it with the standard needle, making it a good alternative for ID vaccination.

Our study has several limitations. Firstly, we failed to meet the sample size to establish non-inferiority in the proportion of participants with seroconversion due to the exclusion of participants with COVID-19. In addition, we had to adapt the definition of seroconversion rate to the MNA we used in our study, resulting in a different, very strict cut-off. Thirdly, our cohort consisted of young, healthy individuals, limiting generalizability to older individuals. Fourthly, participants were not blinded to allocation, which could have introduced bias in AE reporting. Lastly, we analyzed cellular results in a subgroup of 50 participants, two of whom were unknowingly exposed to SARS-CoV-2 without detectable SARS-CoV-2 anti-S or anti-N IgG at inclusion. There is also a possibility that other participants not included in the subgroup were also pre-exposed. We believe that randomization balanced the distribution of pre-exposed participants across the study groups.

In conclusion, our data support reducing the dose to 1/5th of the mRNA-1273 vaccine, administered intradermally, in terms of immunogenicity and safety, despite somewhat lower neutralizing antibody concentrations. Sero-epidemiological studies suggest that even with reduced efficacy against symptomatic infection, fractional dose vaccination could still provide high levels of protection against severe disease on the population level through increased availability (and speed) of vaccination. This would ultimately reduce total infections and death, compared to a scenario where more people remain unvaccinated for a longer period^26^. As such, fractional dose mRNA-1273 vaccine delivered intradermally could have important public health and economic benefits, with fewer side effects and minor loss of efficacy, making it a preferable option for achieving herd immunity quickly. Currently, with high vaccination rates and fewer severe cases due to the decreased severity of the Omicron variant in combination with pre-existent immunity, vaccine coverage is less urgent. However, in case of the emergence of a new, more virulent VOC, boosting with a new vaccine does become more urgent as there will be high and fast vaccine coverage. Therefore, in future pandemics, it would be advisable to evaluate dose-sparing fractional ID doses versus full-dose priming regimens early on during drug development.

## Methods

### Study design

We performed an open-label, randomized controlled trial at the vaccination clinic of the Leiden University Medical Center (LUMC), a tertiary referral hospital in the Netherlands, in collaboration with the Center for Human Drug Research (CHDR), Leiden, The Netherlands. The trial was approved by the Medical Ethical Committee Leiden-Den Haag-Delft and registered in the International Clinical Trials Registry Platform (EUCTR2021-000454-26-NL). The study was done in accordance with Good Clinical Practice Guidelines and monitored by an independent Data and Safety Monitoring Board.

### Participants

Healthy adults between 18 and 30 years and without a history of laboratory-confirmed or self-reported SARS-CoV-2 infection were eligible. Other main exclusion criteria were prior SARS-CoV-2 vaccination, immunodeficiency or autoimmune disease, use of corticosteroids, and pregnancy (see the protocol for a full list). All participants provided written informed consent before enrollment.

### Randomization and masking

Participants were randomized by block randomization in a 1:1:1 ratio to receive either a fractional dose of 20 µg mRNA-1273 ID through a standard needle (ID-SN) or through the Bella-mu® 1.4 mm microneedle (ID-BM) or standard dose of 100 µg mRNA-1273 vaccine IM. Participants and investigators were aware of allocation, given the different routes of administration. Laboratory personnel assessing outcomes were blinded to allocation.

### Procedures

The vaccine was prepared according to the manufacturer’s instructions. At day one (D01), a 1/5th ID dose of 0.1 mL was injected in the deltoid region with a standard needle and syringe (Becton Dickinson U-100 Micro-Fine insulin syringes with integrated 29 G needle) or with a Bella-mu® 1.4 mm microneedle. The standard needle was inserted at a 5-to-15-degree angle and advanced approximately 3 mm through the epidermis to ensure that the entire bevel was covered by the skin using the Mantoux technique^27^. The Bella-mu® 1.4 mm microneedle was placed perpendicularly onto the skin until the hub loosely touched the surface of the skin, and then the vaccine was injected at a controlled depth of about 1 mm. After each ID injection, a wheal appeared on the skin, which was quantified in mm as a quality indicator of the vaccination technique, with a cut-off diameter of 6 mm or more^28^. Participants in the IM group received the standard dose of 0.5 mL in the deltoid muscle. The second dose was administered on the contralateral side.

Participants were followed up by telephone calls on days 2, 4, 8, and 15 after each vaccination and by on-site visits on day 29 (D29), day 36, day 43, and month seven (M07). Participants recorded the nature and severity of any (un)solicited local and systemic AE and the use of medication in a diary up to 14 days following each vaccination (Supplement D). All AEs were assessed according to a standardized grading scale (Supplementary Tables 1–3) and to the International Classification of Disease-10 (ICD-10) terms. Stopping rules were applied in case any grade 4 AE occurred or a grade 3 AE was reported more than once (Supplement B).

We collected blood samples at D01 and at each scheduled on-site follow-up visit. Serum samples were separated, aliquoted, and stored at −80 °C until analysis.

### Immunogenicity

SARS-CoV-2-spike-S1 and -RBD IgG-binding antibodies in serum were measured by a bead-based multiplex immunoassay (MIA) based on Luminex technology^29,30^. Antibody concentrations were interpolated using a 5-parameter fit of a serum pool calibrated against the WHO international reference (NIBSC, no 20/136) and reported in binding antibody units per mL (BAU/mL)^29^. Seropositivity was defined as a SARS-CoV-2-spike-S1 and -RBD antibody concentration of more than 10 and more than 30 BAU/mL, respectively^19^.

We measured neutralizing antibody concentrations against SARS-CoV-2 D614G by micro-neutralization assay (MNA), as previously described^31^. In short, heat-inactivated serum samples were diluted two-fold in a 96-well plate, and 75 µl/well of diluted wild-type SARS-CoV-2 virus was added. After 1 h incubation at 37 °C, the virus-antibody mixture was added to Vero E6 cells [ECACC, cat. No. 85020206]. After overnight incubation at 37 °C, cells were fixed with formaldehyde. Virus-infected foci were visualized by SARS-CoV-2 immunostaining [ImmunoSpot S6 Ultra-V analyzer with BioSpot counting module (Cellular Technologies Europe)], and foci were counted with SoftMax Pro [Molecular Devices, cat. no. SMP7X GXP SINGLE COMP or SMP7X GXP SERVER]. Neutralization titer was expressed as ND50, i.e., the serum dilution at which infection of Vero E6 cells was reduced by 50%, compared to the positive control. Neutralizing titers of the serum samples were also calibrated against an international reference serum (1st WHO International Standard for anti-SARS-CoV2 antibody (20/136))^32^ and are reported in IU/mL. The lower limit of detection was 15.25 IU/mL.

In a subgroup of participants from the ID-SN (*n* = 26) and IM group (*n* = 24), we collected additional blood samples and isolated peripheral blood mononuclear cells (PBMC) to perform an in-depth analysis of T- and B-cell responses to SARS-CoV-2 antigens. The analysis of these immune responses is described in the Supplementary Appendix (Supplements E and F). Briefly, immunophenotyping of SARS-CoV-2-spike protein-specific B-cells was performed by flow cytometry. Spike-specific T-cells were detected by flow cytometry using peptide stimulation followed by intracellular (cytokine) staining and, in parallel, peptide-HLA tetramer technology.

### Intercurrent COVID-19 infection

Before enrollment and at every study visit, participants were screened for SARS-CoV-2 infection by serology [SARS-CoV-2 anti-nucleocapsid [anti-N] IgG antibodies (Alinity m SARS-CoV-2 assay, Abbot Molecular, IL, USA) and MIA] and SARS-CoV-2 PCR of a mid-turbinate/ throat swab. Participants who tested positive were withdrawn from the study.

### Outcomes

The primary outcome was non-inferiority in the proportion of participants with seroconversion, as determined by 50% virus neutralization, measured on D43 after vaccination for fractional dose ID (SN or BM) compared with standard dose IM. Seroconversion was defined as a post-vaccination rise in neutralizing antibody concentration of at least 465 IU/mL, which was the lowest concentration measured in the IM group. Safety was also a primary outcome and included the nature and severity of local and systemic related AE up to 14 days after each vaccination. Secondary outcomes included geometric mean concentrations (GMC) of binding and neutralizing antibodies at D01, D29, D36, and M07 and geometric mean fold rise (GMFR) between consecutive time points.

### Statistical analysis

For the primary endpoint analysis, a non-inferiority margin of 15% was set for the difference in response between the fractional ID doses and the standard IM dose. We based the sample size on the phase-1 dose-escalation study of Jackson et al.^15^. We assumed >90% seroconversion after the standard IM dose and considered that reduction to 75% seroconversion with fractional ID dose would still provide sufficient protection against severe disease on a population scale^10^. Based on these assumptions, we defined seroconversion as an antibody titer of ≥128, measured by an 80% plaque reduction test (PRNT_80_). A sample size of 55 participants per study group was required to detect a non-inferiority margin of 15%, with 80% power, 5% significance level for a one-sided test, and accounting for 10% loss to follow-up. In total, 165 participants were to be recruited.

We compared the ID fractional dose (ID-SN and ID-BM) groups pairwise with the standard IM dose in an intention-to-treat (ITT) population, which included all eligible randomized participants who were seronegative at baseline and who remained negative for SARS-CoV-2 anti-N IgG-binding antibodies during the study, with at least one valid antibody test result.

Neutralizing antibodies were expressed as GMC, geometric mean titers (GMT; Supplements), and GMFR with corresponding 95% geometric confidence interval (CI). Any ND_50_ concentration reported as seronegative (limit of quantification [LOQ] < 15.3) was converted to LOQ/2. Non-inferiority was demonstrated if the lower bound of the two-sided 95% CI for the seroconversion rate difference between the ID and IM groups was smaller than 15%. GMFR was calculated as the mean of the difference of logarithmically transformed test results (later time point minus earlier time point) and transformed back to the original scale. Levels of IgG-binding antibodies against SARS-CoV-2-spike-S1 and -RBD were expressed as GMC with a 2-sided 95% geometric CI. To enable ratio calculation for the GMFR for D29/D01, D43/D01, and M07/D01, any SARS-CoV-2-spike-S1 and -RBD antibody concentration at D01 reported below 1 was set to 1.

mRNA-1273-induced T-cell responses were analyzed in the ITT subgroup population. B-cell responses to SARS-CoV-2 antigens were assessed in the per-protocol (PP) and ITT populations. The ITT population included all participants in the subgroup from the ID-SN (*n* = 26) and IM group (*n* = 24), whereas the PP population excluded participants in the subgroup who had SARS-CoV-2 specific B-cells at baseline.

Safety outcomes were assessed in the ITT population, including all randomized participants who received at least one dose of mRNA-1273 vaccine, including those with COVID-19 illness. The safety endpoints, except wheal diameter, are presented as counts and percentages. Wheal diameter was reported as median with interquartile range.

Statistical analyses were done using IBM SPSS Statistics for Windows, version 25.0. Armonk, New York: IBM Corp. Graphs were made using Graphpad version 9.3.1 for Windows, GraphPad Software, San Diego. California.

### Reporting summary

Further information on research design is available in the Nature Research Reporting Summary linked to this article.

### Supplementary information

Supplements

REPORTING SUMMARY

## Data Availability

The datasets used and analyzed during the current study are available from the corresponding author upon reasonable request.
