# Supplementary material for: Immunogenicity and reactogenicity of intradermal mRNA-1273 SARS-CoV-2 vaccination: a non-inferiority, randomized-controlled trial
Source: NPJ Vaccines. 2024 Jan 2;9:1. doi: 10.1038/s41541-023-00785-w (PMC10761693; doi:10.1038/s41541-023-00785-w)
Supplement: Supplementary file 1 — Supplements [file 41541_2023_785_MOESM1_ESM.pdf]

Supplements to:

Tolerability, safety and immunogenicity of intradermal delivery of a fractional dose mRNA-1273 SARS-CoV-2 vaccine in healthy adults as a dose sparing primary vaccination strategy: a non-inferiority, randomized-controlled trial

Manon L.M. Prins, M.D.[1], Geert V.T. Roozen, M.D.[1,2], Cilia. R. Pothast, MSc[3], Wesley Huisman, MSc[2], Rob van Binnendijk, Ph.D.[4], Gerco den Hartog, Ph.D.[4,6], Vincent P. Kuiper, M.D.[2], Corine Prins, BSc[1], Jacqueline J. Janse, MSc[2], Olivia. A.C. Lamers, M.D.[2], Jan Pieter R. Koopman, M.D.[2], Annelieke C. Kruithof, Ph.D.[1,5], Ingrid M.C. Kamerling, PhD[1,5], Romy C. Dijkland[3], Alicia. C de Kroon[2], Shohreh Azimi[2], Mariet C.W. Feltkamp, M.D., Ph.D.[7], Marjan Kuijer[4], Simon P. Jochems, Ph.D.[2], Mirjam H.M. Heemskerk, Ph.D.[3], Frits R. Rosendaal, M.D., Ph.D.[8], Meta Roestenberg, M.D., Ph.D.[1,2], Leo G. Visser, M.D., Ph.D.[1], Anna H.E. Roukens, M.D., Ph.D.[1]

1. Department of Infectious Diseases, Leiden University Medical Center, Leiden, The Netherlands
2. Department of Parasitology, Leiden University Medical Center, Leiden, The Netherlands
3. Department of Haematology, Leiden University Medical Center, Leiden, The Netherlands
4. Department of Immune Surveillance, National Institute for Public Health and the Environment, The Netherlands
5. Center for Human Drug Research, Leiden, The Netherlands
6. Laboratory of Medical Immunology, RadboudUMC, Nijmegen, The Netherlands
7. Department of Medical Microbiology, Leiden University Medical Center, Leiden, The Netherlands
8. Department of Clinical Epidemiology, Leiden University Medical Center, Leiden, The Netherlands

A. Full list of investigators and contributors
B. Prespecified stopping rules
C. Solicited adverse events
Supplementary Table 1. Grading scale for severity of solicited vaccine-related local adverse reactions
Supplementary Table 2. Grading scale for severity of solicited vaccine-related systemic adverse reactions
Supplementary Table 3. Grading scale for severity of vaccine-related fever
D. Supplementary Figure 1. Participants diary for the registration of adverse events and concomitant medication use
E. Supplementary Methods- Additional immunologic B-cell assay method details
Supplementary Table 4. Panel of different monoclonal antibodies used for phenotypical analysis of SARS-CoV-2 specific B-cells.
Supplementary Figure 2. Gating strategy to detect SARS-CoV-2-spike-specific B-cells.
F. Supplementary Methods- Additional T-cell immunity assay method details
Supplementary Table 5. List of peptides
Supplementary Table 6. List of antibodies and reagents used for flow cytometry
Supplementary Table 7. List of peptide-HLA tetramers used for flow cytometry
Supplementary Figure 3. Flow cytometry gating example for peptide stimulation assay and tetramer staining
G. Supplementary Table 8. Characteristics of participants at inclusion.
H. Antibody response
Supplementary Figure 4. Geometric mean concentrations of anti-RBD and anti-N and geometric mean fold rise of anti-RBD IgG in BAU/mL (95% CI).
Supplementary Table 9. Antibody concentrations reported in GMCs (IgG and neutralization) and GMTs (neutralization).
Supplementary Table 10. Fold change increase of GMC (IgG) and GMTs (neutralization), reported in GMFR.
I. SARS-CoV-2 specific B-cell response
Supplementary Figure 5. Participants with SARS-CoV-2-spike-specific B-cells prior to vaccination.
Supplementary Figure 6. B-cell compartment and the immunogenicity of intradermal and intramuscular delivery of mRNA SARS-CoV-2 vaccine
Supplementary Figure 7. Gating strategy of class-switching of SARS-CoV-2-specific B-cells.
Supplementary Figure 8. Class-switching of participants with SARS-CoV-2-spike-specific B-cells prior to vaccination.
Supplementary Figure 9. Uniform Manifold Approximation and Projection (UMAP) of all SARS-CoV-2-specific B-cells.
J. SARS-CoV-2 specific T-cell responses
Supplementary Figure 10. mRNA-1273 induced SARS-CoV-2-specific T-cell responses
K. Adverse events.
Supplementary Table 11. Number of participants who used antipyretics for related adverse events.
Supplementary Table 12. Local and systemic adverse events, related to the vaccination.
Supplementary Table 13. Adverse events related to vaccine administration after vaccination one and two.
Supplementary Table 14. Numbers of participants with recurrence of erythema and swelling >2.5 cm after vaccination one and two.
Supplementary Figure 11. Recurrence of erythema and swelling.
Supplementary Table 15. Number of participants that reported a severe adverse event.
Supplementary Table 16. Numbers of severe adverse events reported by the participants.

Supplement A: Full list of Investigators and Contributors

The following individuals constitute the IDSCOVA Study Group (*indicates principal investigator):

Leiden University Medical Center: M.L.M. Prins, G.V.T. Roozen, C.R. Pothast, W. Huisman, J.J. Janse, C. Prins, R.C. Dijkland, A. de Kroon, S. Azimi, C. Crul, P.H. Verbeek, O.A.C. Lamers, J.P.R. Koopman, A. Esmeijer, J. Zang, K.J.M. Reuvekamp, M.C.W. Feltkamp, F.R. Rosendaal, M. Roestenberg, L.G. Visser, A.H.E. Roukens (*),

Center for Human Drug Research: A.C. Kruithof

National Institute for Public Health and the Environment: R. van Binnendijk, G. den Hartog, M. Kuijer

Supplement B: Prespecified stopping rules

The following stopping rules were in place for all participants, based on review of diary reactogenicity and AE data.

Stopping Rule Criteria Safety:

- If any participant vaccinated with mRNA-1273 (at any dose level) develops an SAE or SUSAR that is assessed by the investigator as possibly related, or for which there is no alternative, plausible, attributable cause.
- If any participant vaccinated with mRNA-1273 (at any dose level) develops a Grade 4 local reaction within 14 days after vaccination that is assessed as possibly related by the investigator, or for which there is no alternative, plausible, attributable cause.
- If any participant vaccinated with mRNA-1273 (at any dose level) develops a Grade 4 systemic event within 7 days after vaccination that is assessed as possibly related by the investigator, or for which there is no alternative, plausible, attributable cause
- If two participants vaccinated with mRNA-1273 (within the same dose level) develop a fever >40.0°C for at least one daily measurement within 7 days after vaccination that is assessed as possibly related by the investigator, or for which there is no alternative, plausible, attributable cause.
- If any two participants vaccinated with mRNA-1273 (at any dose level) report the same or similar severe (Grade 3) AE within 14 days after vaccination, assessed as possibly related by the investigator, or for which there is no alternative, plausible, attributable cause.
- If any participant dies or requires ICU admission due to SARS-CoV-2 infection; if this stopping rule is met, all available clinical and preclinical safety and immunogenicity data should be reviewed to evaluate for enhanced COVID-19 disease.

Stopping Rule Criteria Immunogenicity:

- If <12/15 participants vaccinated with mRNA-1273 (at 20µg dose level) do not meet the threshold for neutralizing antibodies (PRNT80 ≥128) at Day 43.
- If part 2 is unsafe and ≤7 out of 10 participants of part 1 developed virus neutralizing antibody titers (PRNT80) ≥128 at day 43,

The anti-Spike IgG antibody titers may be used as a proxy for the neutralizing antibody titers if the results of the neutralizing antibody assay are delayed.

Supplement C: Solicited adverse events

Local reactions

Redness and swelling were measured and recorded in centimeters and categorized as absent, mild, moderate, or severe based on the grading scale (Supplementary Table 1).

Pain at the injection site was assessed by the participant as absent, mild, moderate, or severe according the grading scale in Supplementary Table 1.

Systemic events

Solicited systemic events consisted of vomiting, diarrhoea, headache, fatigue, chills, new or worsened muscle pain, and new or worsened joint pain. The symptoms were assessed by the participant as absent, mild, moderate, or severe according to the grading scale in Supplementary Table 2.

Fever

Participants were instructed on how to measure oral temperature at home. Daily temperature measurements were registered in the diary in the morning and at any time when fever is suspected during the diary data collection periods. Fever was defined as an oral temperature of ≥38.0°C. The highest temperature for each day was recorded in the diary. Temperature was measured and recorded to 1 decimal place and then categorized during analysis according to the scale shown in Supplementary Table 3.

Supplementary Table 1. Grading scale for severity of solicited vaccine-related local adverse reactions

	**Mild (Grade 1)**	**Moderate (Grade 2)**	**Severe (Grade 3)**	**Potentially life threatening (Grade 4)**
**Pain at injection site**	Does not interfere with activity	Interferes with activity	Prevents daily activity	Emergency room visit or hospitalization for severe pain
**Redness**	2.5 cm to 5.0 cm	>5.0 cm to 10.0 cm	>10 cm	Necrosis or exfoliative dermatitis
**Swelling**	2.5 cm to 5.0 cm	>5.0 cm to 10.0 cm	>10 cm	Necrosis
**Pain and swelling at regional lymph nodes**	Does not interfere with activity	Interferes with activity	Prevents daily activity	Emergency room visit or hospitalization for severe pain

Supplementary Table 2. Grading scale for severity of solicited vaccine-related systemic adverse reactions

	**Mild (Grade 1)**	**Moderate (Grade 2)**	**Severe (Grade 3)**	**Potentially life threatening (Grade 4)**
**Vomiting**	1-2 times in 24 hours	>2 times in 24 hours	Requires IV hydration	Emergency room visit or hospitalization for hypotensive shock
**Diarrhoea**	2 to 3 loose stools in 24 hours	4 to 5 loose stools in 24 hours	6 or more loose stools in 24 hours	Emergency room visit or hospitalization for severe diarrhoea
**Headache**	Does not interfere with activity	Some interference with activity	Prevents daily routine activity	Emergency room visit or hospitalization for severe headache
**Fatigue/tiredness**	Does not interfere with activity	Some interference with activity	Prevents daily routine activity	Emergency room visit or hospitalization for severe fatigue
**Chills**	Does not interfere with activity	Some interference with activity	Prevents daily routine activity	Emergency room visit or hospitalization for severe chills
**New or worsened muscle pain**	Does not interfere with activity	Some interference with activity	Prevents daily routine activity	Emergency room visit or hospitalization for severe new or worsened muscle pain
**New or worsened joint pain**	Does not interfere with activity	Some interference with activity	Prevents daily routine activity	Emergency room visit or hospitalization for severe new or worsened joint pain

Supplementary Table 3. Grading scale for severity of vaccine-related fever

**Absent**	<38.0°C
**Mild**	38.0-38.4°C
**Moderate**	38.5-38.9°C

**Severe**	39.0-40.0°C
**Very severe**	>40.0°C

Supplement D: Supplementary Figure 1. Participants diary for the registration of adverse events and concomitant medication use

DAY 1:

<table>
<tr><td>DATE DIARY:

|__|__| |__|__| |__|__|__|__|</td><td>TIME OF FILLING IN DIARY:

|__|__| : |__|__|</td><td>NUMBER OF PARTICIPANT: IDSCOVA

|__|__|__|</td></tr>
</table>

Please circle the answer that best applies to you or write a short comment:

<table>
<tr><th>LOCAL REACTIONS:</th><th>Absent</th><th>Mild</th><th>Moderate</th><th>Severe</th><th>Potentially life threatening</th></tr>
<tr><td>Pain at injection site</td><td>None</td><td>Does not interfere with daily activities</td><td>Interferes with daily activities</td><td>Severe pain</td><td>Emergency room visit or hospitalisation for severe pain</td></tr>
<tr><td>Muscle stiffness</td><td>None</td><td>Does not interfere with daily activities</td><td>Interferes with daily activities</td><td>Prevents daily activitie</td><td>Emergency room visit or hospitalisation for severe muscle stiffness</td></tr>
<tr><td>Redness</td><td>|__|__|cm</td><td>|__|__|cm</td><td>|__|__|cm</td><td>|__|__|cm</td><td>|__|__|cm</td></tr>
<tr><td></td><td><2.5 cm</td><td>2.5 cm tot 5.0 cm</td><td>>5.0 cm tot 10 cm</td><td>> 10 cm</td><td>Necrosis or exfoliative dermatitis</td></tr>
<tr><td>Swelling</td><td>|__|__|cm</td><td>|__|__|cm</td><td>|__|__|cm</td><td>|__|__|cm</td><td>|__|__|cm</td></tr>
<tr><td></td><td><2.5 cm</td><td>2.5 cm tot 5.0 cm</td><td>>5.0 cm tot 10 cm</td><td>> 10 cm</td><td>Necrosis</td></tr>
<tr><td>Pain and swelling at regional lymph nodes</td><td>None</td><td>Does not interfere with daily activities</td><td>Interferes with daily activities</td><td>Prevents daily activities</td><td>Emergency room visit or hospitalisation for severe pain</td></tr>
<tr><td></td><td></td><td></td><td></td><td></td><td></td></tr>
<tr><th>SYSTEMIC REACTIONS:</th><th>Absent</th><th>Mild</th><th>Moderate</th><th>Severe</th><th>Potentially life threatening</th></tr>
<tr><td>Vomiting</td><td>None</td><td>1-2 times in 24 hours</td><td>>2 times in 24 hours</td><td>Requires IV hydration</td><td>Emergency room visit or hospitalisation for hyptensive shock</td></tr>
<tr><td>Dhiarrhoea</td><td>None</td><td>2 to 3 loose stools in 24 hours</td><td>4 to 5 loose stools in 24 hours</td><td>6 or more loose stools in 24 hours</td><td>Emergency room visit or hospitalisation for severe diarrhoea</td></tr>
<tr><td>Headache</td><td>None</td><td>Does not interfere with daily activities</td><td>Some interference with daily activities</td><td>Prevents daily activities</td><td>Emergency room visit or hospitalisation for severe headache</td></tr>
<tr><td>Fatigue/ tiredness</td><td>None</td><td>Does not interfere with daily activities</td><td>Some interference with daily activities</td><td>Prevents daily activities</td><td>Emergency room visit or hospitalisation for severe fatigue</td></tr>
<tr><td>Chills</td><td>None</td><td>Does not interfere with daily activities</td><td>Some interference with daily activities</td><td>Prevents daily activities</td><td>Emergency room visit or hospitalisation for severe chills</td></tr>
<tr><td>New or worsened muscle pain</td><td>None</td><td>Does not interfere with daily activities</td><td>Some interference with daily activities</td><td>Prevents daily activities</td><td>Emergency room visit or hospitalisation for severe muscle pain</td></tr>
<tr><td>New or worsened joint pain</td><td>None</td><td>Does not interfere with daily activities</td><td>Some interference with daily activities</td><td>Prevents daily activities</td><td>Emergency room visit or hospitalisation for severe joint pain</td></tr>
</table>

<table>
<tr><td>DAY 1: DATE DIARY
|__|__| |__|__| |__|__|__|__|</td><td>TIME OF FILLING IN DIARY:
|__|__| : |__|__|</td><td>PARTICIPANT NUMBER: IDSCOVA
|__|__|__|</td></tr>
</table>

<table>
<tr><td>Temperature</td><td>Absent</td><td>Mild</td><td>Moderate</td><td>Severe</td><td>Very severe</td></tr>
<tr><td>|__|__|__|</td><td><38°C</td><td>38.0°C – 38.4°C</td><td>38.5°C -38.9°C</td><td>39.0°C -40.0°C</td><td>>40.0°C</td></tr>
</table>

<table>
<tr><td>New concomitant medication (date)</td><td>Name medication</td><td>Dose & units</td><td>How many times a day</td><td>Indication</td><td>By general practitioner?</td></tr>
<tr><td>from:
|__|__| |__|__| |__|__|
to:
|__|__| |__|__| |__|__|</td><td></td><td>______</td><td>|__|__|a day</td><td></td><td>|__| YES
|__| NO</td></tr>
<tr><td>from:
|__|__| |__|__| |__|__|
to:
|__|__| |__|__| |__|__|</td><td></td><td>______</td><td>|__|__|a day</td><td></td><td>|__| YES
|__| NO</td></tr>
<tr><td>from:
|__|__| |__|__| |__|__|
to:
|__|__| |__|__| |__|__|</td><td></td><td>______</td><td>|__|__|a day</td><td></td><td>|__| YES
|__| NO</td></tr>
<tr><td>from:
|__|__| |__|__| |__|__|
to:
|__|__| |__|__| |__|__|</td><td></td><td>______</td><td>|__|__|a day</td><td></td><td>|__| YES
|__| NO</td></tr>
<tr><td>from:
|__|__| |__|__| |__|__|
to:
|__|__| |__|__| |__|__|</td><td></td><td>______</td><td>|__|__|a day</td><td></td><td>|__| YES
|__| NO</td></tr>
<tr><td>from:
|__|__| |__|__| |__|__|
to:
|__|__| |__|__| |__|__|</td><td></td><td>______</td><td>|__|__|a day</td><td></td><td>|__| YES
|__| NO</td></tr>
<tr><td>Other (systemic) reactions:</td><td colspan="5">Remarks:</td></tr>
</table>

Diary for the solicited adverse events and concomitant medication. Participants were instructed to record their temperature and adverse events daily for fourteen days after each vaccination

Supplement E: Supplementary Methods- Additional immunologic B-cell assay method details

Analysis of SARS-CoV-2-spike-specific B-cells using spectral flow cytometry

For immunophenotyping of SARS-CoV-2-specific B-cells, $1x10^7$ PBMCs were thawed in pre-warmed RPMI (Gibco) containing 20% heat-inactivated Fetal Bovin Serum (FBS; Lonza) and washed once in the same media. All centrifugation steps of non-fixed samples were performed at 450g. Cells were transferred to a 96-wells well, washed with PBS and then resuspended in 100 µl PBS containing 1/500 diluted LIVE/DEAD fixable Blue Dead Cell Stain (Invitrogen Cat#L23105) and 1/50 diluted Fc Receptor binding inhibitor (eBioscience cat# 14-9161-73) for 15 minutes at room temperature (RT) in the dark. Then, samples were washed with PBS containing 2 mM EDTA and 0.5% BSA followed by resuspension in 100 µl of extracellular antibody cocktail (see Supplementary Table 4) for 15 minutes at RT in the dark (see below). This cocktail includes streptavidin-coupled BV421 (BD, cat#563259) as a 'decoy probe' to remove B-cells that react with streptavidin. Then stained samples were resuspended into 100 µl of 2 µg/ml of BUV615-labeled and BUV661-labeled SARS-CoV-2-spike-specific tetramers for 30 minutes on ice in the dark. For the intracellular antibody staining, cells were first resuspended in FoxP3 fixation/permeabilization solution (Invitrogen, cat#00-5521-00) and incubated for 30 minutes on ice in the dark, followed by three washes with 1x permeabilization Buffer (Invitrogen, cat#00-8333-56). All centriguation steps of fixed samples were performed at 800g. Then, samples were resuspended in intracellular antibody cocktail, containing Ki67-BV711 (Biolegend, cat#350515), IRF4-APC (Miltenyi, cat#130-100-915) and caspase-3-V450 (BD, cat#560627). Fixed cells were washed with PBS containing 2 mM EDTA and 0.5% BSA and $3\text{-}5x10^6$ events were acquired on a Cytek Aurora 5L spectral flow cytometer and unmixed using SpectroFlo software. See Supplementary Table 4 for details on the type of single-stain reference controls used for unmixing. FLOWjo software was used to gate CD19+ B-cells and OMIQ data analysis software was used for further analysis (www.omiq.ai). Cytonorm was used for batch corrections followed by Uniform Manifold Approximation and Projection (UMAP) dimensionality reduction to visualize the phenotypes of SARS-CoV-2-spike-specific B-cells. For gating strategies for SARS-CoV-2-specific B-cells see Supplementary Figure 2.

Generation of SARS-CoV-2 spike labelled tetramers

Fluorescently labelled tetramers were generated by labeling biotinylated SARS-CoV-2 spike protein (R&D, cat#10549-050) with streptavidin-coupled BUV615 (BD, cat#613013) or streptavidin-coupled BUV661 (BD, cat#612979). SARS-CoV-2 spike protein was resuspended in 100 ul PBS to get a 500 ug/ml stock mixture (3730 pmol/ml). SARS-CoV-2 spike protein was then diluted two times to get a 1865 pmol/ml user solution. Biotinylated SARS-CoV-2 spike protein was then mixed in an approximate 4:1 molecular ratio with each of the 2 streptavidin-coupled fluorochromes (at 100 ug/ml). Incubation was performed on ice in a stepwise approach where $1/10^{th}$ fraction of streptavidin-coupled fluorochrome was added to the biotinylated SARS-CoV-2-spike protein every 10 minutes. At 50 and 100 minutes the tetramers were spun down using a short pulse-spin. After the final incubation, free biotin was added to a final concentration of 30 uM and incubated for 30 min on ice to block all unbound streptavidin-coupled fluorochromes. Labeled SARS-CoV-2 spike tetramers (91 ug/ml) were stored in the dark at $4^{O}C$ and used within 2 weeks.

Supplementary Table 4. Panel of different monoclonal antibodies used for phenotypical analysis of SARS-CoV-2-specific B-cells.

	Marker	Fluorochrome	Clone	Company	Catalogue	Dilution	Ref control
1	CD20	Pacific orange	HI47	ThermoFisher	MHCD2030	20	Cells
2	CD138	BUV737	MI15	BD	612834	50	Beads
3	CD56	BV510	HCD56	Biolegend	318340	50	Cells
4	CD10	BV605	HI 10 a	Biolegend	312222	100	Beads
5	CD27	APC-R700	M-T271	BD	565116	100	Cells
6	CD38	APC-Fire810	HIT2	Biolegend	303550	100	Cells
7	PD-1	BV480	EH12.1	BD	566112	100	Beads
8	caspase-3	V450	C92-605	BD	560627	100	Beads
9	CD3	BV510	OKT3	Biolegend	317332	100	Cells
10	CD19	BUV395	HIB19	BD	740287	200	Cells
11	CD21	BUV805	BLy4	BD	742008	200	Cells
12	CD5	BV750	L17F12	BD	747090	200	Cells
13	CD73	AF647	AD2	Abcam	243083	200	Cells
14	CCR10	BB515	1B5	BD	564769	200	Cells
15	IRF4	APC	REA201	Miltenyi	130-100-915	200	Cells
16	HLA-DR	BUV496	G46-6	BD	749866	200	Cells
17	IgD	BUV563	I-A6-2	BD	741394	400	Cells
18	IgM	BV570	MHM-88	Biolegend	314517	400	Cells
19	IgG	PE-CF594	G18-145	BD	562538	400	Cells
20	CD95	PE-Cy5	DX2	Biolegend	305610	400	Cells
21	CD43	PerCP-Cy5.5	1G10	BD	563521	400	Cells
22	CD45RB	PE	MEM-55	Biolegend	310204	400	Cells
23	CD11c	BV650	Bu15	Biolegend	337237	400	Cells
24	Ki67	BV711	Ki67	Biolegend	350515	400	Cells
25	Live/Dead	Blue		ThermoFisher	L23105	500	Cells
26	CXCR3	PE-Cy7	G025H7	Biolegend	353719	1200	Cells
27	IgA	APC-Vio770	IS11-8E10	Miltenyi	130-113-999	1600	Cells

Supplementary Figure 2. Gating strategy to detect SARS-CoV-2-spike-specific B-cells.

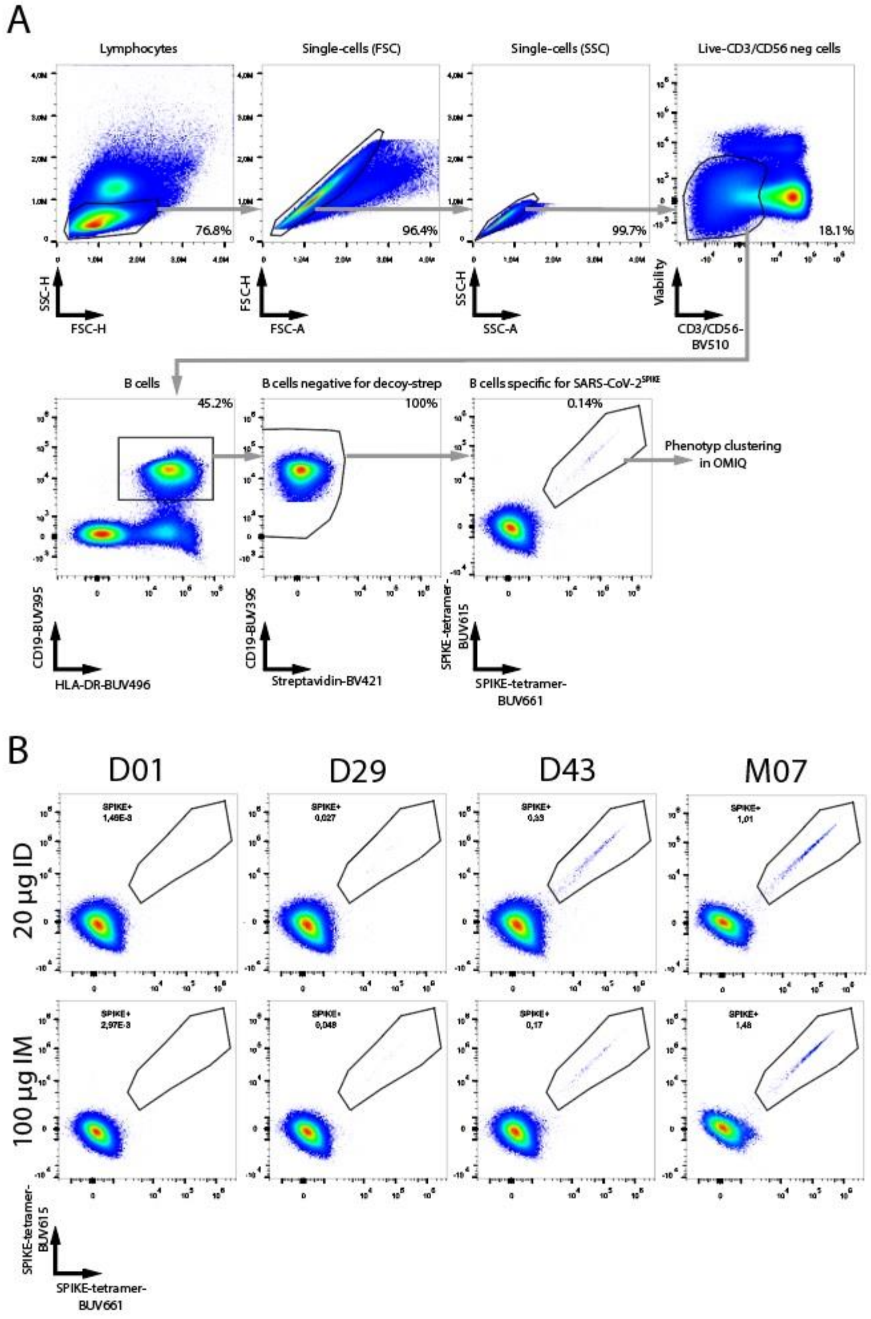

For immunophenotyping of SARS-CoV-2-specific B-cells, Peripheral Blood Mononuclear Cells (PBMCs) were thawed and stained with a cocktail of antibodies targetting extracellulair proteins and SARS-CoV-2 spike-labeled tetramers. **A**. Single-cell lymphocytes were first gated followed by exclusion of T and NK lymphocytes (CD3/CD56). Next, B-cells were gated (CD19) followed by exclusion of B-cells that were binding to streptavidin complexes that were not labeled with SARS-CoV-2 (decoy). SARS-CoV-2-spike-specific B-cells were gated based on double positivity for SARS-CoV-2 spike-labeled tetramers (BUV661 and BUV615). **B.** Gated SARS-CoV-2-spike-specific B-cells are shown for representative samples from ID (top panel) and IM (bottom panel) vaccine deliveries over time.
ID=intradermal; IM=intramuscular; D=day; M=month

Supplement F: Supplementary Methods- Additional T-cell immunity assay method details

Analysis of SARS-CoV-2-spike-specific T-cells using flow cytometry

In addition, we obtained PBMCs at day 1, 29, 43 and month 7 after ID and IM administration. PBMCs were isolated from fresh whole blood using Ficoll-Isopaque and cryopreserved until further use. Upon thawing, the PBMCs were slowly diluted in culture medium consisting of Iscove Modified Dulbecco Medium (IMDM; Lonza) supplemented with 10% heat-inactivated fetal bovine serum (FBS; Sigma-Aldrich), 2.7 mM L-glutamine (Lonza), 100 U/mL penicillin (Lonza) and 100µg/mL streptomycin (Lonza) (1% p/s). After thawing and washing, PBMCs were treated with 1.33 mg/ml DNAse to minimize cell clumping, counted and used for T-cell stimulation assay as well as for peptide-HLA tetramer staining. For the T-cell stimulation assay, up to $2x10^6$ PBMCs were seeded in 100 µL culture medium and stimulated with 15-mer peptides with 11 amino acid overlap which cover the whole SARS-CoV-2 spike antigen (SB peptide, France) in 96-well round bottomed plates. The peptides were dissolved in 20% dimethyl sulfoxide (DMSO) in ddH_2O and added to the wells in a 1 µg/mL final concentration. As a negative control, DMSO and ddH_2O in the same concentration was added. As a positive control, a pool of peptides from CMV, EBV, Flu and extended (CEFX) was dissolved in 20% DMSO in ddH_2O and in a final concentration of 0.25 µg/mL (see Supplementary Table 5 for CEFX peptide pool details). After one hour incubation (37°C, 5% CO_2), 5 µg/mL Brefeldin A was added to the well and the plate was further incubated for 15 hours. The stimulation reaction was stopped by washing the cells in PBS followed by viability staining using Zombie-Red. After a PBS wash, cells were fixated and permeabilized using the FOXP3 buffer kit (Thermo Fisher). 20 µL antibody staining mix was added containing 0.8 mg/mL albumin, Brilliant Stain Buffer Plus and antibodies directed against CD3, CD4, CD8, CD154, CD137, CD69, IFN-γ, TNF-α, IL-2, IL-4, IL-17, PD-1, FOXP3 and CXCR5 (see Supplementary Table 6 for product details). After incubation for 30 minutes at room temperature (RT), the cells were washed in PBS containing 0.8 mg/mL albumin (FACS buffer) and dissolved in 100 µL FACS buffer for measurement on a 3-laser aurora (Cytek Biosciences).

For the tetramer staining, up to $2x10^6$ PBMCs were incubated for 16 hours in 100 µL culture medium. The cells were washed in FACS buffer and stained in two steps. First, 10 µL of an antibody cocktail directed against CD4, CCR7, CD45RA, PD-1 was added together with a tetramer pool in FACS buffer and incubated for 15 minutes at RT. Second, 10 µL of FACS buffer containing CD8 APC-H7 was incubated for an additional 15 minutes at RT (see Supplementary Table 6 for product detail). The tetramer pool contained 23 in-house-made tetramers, consisting of spike peptides and HLAs, conjugated to PE as well as APC (see Supplementary Table 7 for product detail). Cells were washed and dissolved in 100 µL FACS buffer for measurement on a 3-laser aurora (Cytek Biosciences).

For flow cytometry analysis, OMIQ (www.omiq.ai) was used to set gates and retrieve percentages (see Supplementary Figure 5 and S3 for gating strategy). Due to optimal reference controls used for unmixing on the 3-laser Cytek Aurora, no compensation was needed. The same gate was applied to all samples within one donor and as much as possible between donors. Percentage SARS-CoV-2 spike reactive $CD4^+$ T-cells was identified as $CD137^+$ and/or $CD154^+$ cells of total $CD4^+$ T-cells, corrected for background in DMSO. Percentage SARS-CoV-2-spike reactive $CD8^+$ T-cells was identified as $CD137^+$ and/or $CD69^+$ of total $CD8^+$ T-cells. The same $CD137^+$ and/or $CD154^+$ or $CD137^+$ and/or $CD69^+$ was applied to all samples unless the background in DMSO was 0.1% or higher. Then the gate was adapted and applied to all samples of that donor.

For the analysis, percentage SARS-CoV-2-spike $CD4^+$ T-cells was removed if there were less than 10.000 events in CD4 gate and percentage SARS-CoV-2-spike $CD8^+$ T-cells was removed from analysis if there were less than 10.000 events in CD8 gate. A threshold was set on 0.05% for frequency $CD154^+$ and/or $CD137^+$ of total $CD4^+$ T-cells,0.005% for frequency $CD137^+$ and/or $CD69^+$ AND IFN-γ^+ and/or TNF-α^+ of total $CD8^+$ T-cells and 0.005% for frequency of spike/HLA tetramer$^+$ of total $CD8^+$ T-cells. Further gating to calculate percentage of spike-specific cells that are IFN-γ, TNF-α, IL-2, IL-4, IL-17, Tfh (T follicular helper cells; $CXCR5^+PD\text{-}1^+$), PD-1 ($CXCR5^-PD\text{-}1^+$) or Treg ($FOXP3^+IFN\text{-}\gamma^-TNF\text{-}\alpha^-$) positive was only done if frequency threshold was met and if there were more than 25 events in the $CD154^+$ and/or $CD137^+$ gate for $CD4^+$ T-cells and $CD137^+$ and/or $CD69^+$ for the $CD8^+$ T-cells. Percentage of naïve (CCR7+CD45RA+), central memory (CM; CCR7+CD45RA-), effector memory (EM; CCR7-CD45RA-) or terminal effector memory (TEMRA; CCR7-CD45RA+) was measured of spike/HLA-tetramer$^+$ $CD8^+$ T-cells and only calculated if the frequency of spike/HLA-tetramer$^+$ $CD8^+$ T-cells was above threshold and contained at least 10 events. The percentages were exported and further analyzed in Graphpad Prism 9.0.1. All timepoints of the same patient were measured simultaneously to minimize technical variance within one patient. Patients were measured and analyzed in random order to minimize technical variance and bias between cohorts. In the last analysis step the data was separated for the different cohorts.

Supplementary Table 5. List of peptides used in this study.

Peptide pools				
**Pathogen**	**Antigen**	**Supplier**	**Catalogue #**	**Peptide characteristics**
CMV	PP65	JPT	Custom-made	15-mer, 11aa overlapping
Pool	Pool	JPT	PM-CEFX-3	15-mer, 11aa overlapping
EBV	BZLF1	JPT	PM-EBV-BZLF1	15-mer, 11aa overlapping
EBV class I	Mix	LUMC	Custom-made	9-mer, known epitopes (see below)
Influenza A	NP1	JPT	N/A	15-mer, 11aa overlapping, NCBI: ABB79814

EBV class I peptide pool				
**Antigen**	**Sequence (aa)**	**HLA restriction**	**Supplier**	**Peptide characteristics**
LMP2	ESEERPPTPY	A*01:01	LUMC	9-mer
BMLF1	GLCTLVAML	A*02:01	LUMC	9-mer
LMP2	CLGGLLTMV	A*02:01	LUMC	9-mer
LMP2	FLYALALLL	A*02:01	LUMC	9-mer
BRLF1	RVRAYTYSK	A*03:01	LUMC	9-mer
EBNA3A	RLRAEAQVK	A*03:01	LUMC	9-mer
EBNA3B	IVTDFSVIK	A*11:01	LUMC	9-mer
EBNA3B	AVFDRKSDAK	A*11:01	LUMC	9-mer
BRLF1	DYCNVLNKEF	A*24:02	LUMC	9-mer
EBNA3A	RPPIFIRRL	B*07:02	LUMC	9-mer
BZLF1	RAKFKQLL	B*08:01	LUMC	9-mer
EBNA3A	QAKWRLQTL	B*08:01	LUMC	9-mer
EBNA3A	FLRGRAYGL	B*08:01	LUMC	9-mer
EBNA3A	YPLHEQHGM	B*35:01	LUMC	9-mer

Supplementary Table 6. List of antibodies and reagents used for flow cytometry.

**Antibodies**				
**Antigen**	**Format**	**Clone ID**	**Supplier**	**Catalogous #**
CD4	BV510	SK3	BD Biosciences	562970
CD8	APC-H7	SK1	BD Biosciences	560179
CD3	Pe-Texas-Red	7D6	Invitrogen	MHCD0317
CD69	FITC	Clone L78	BD Biosciences	347823
CD137	APC	4B4-1	BD Biosciences	550890
CD154	Pacific blue	24-31	Biolegend	310820
IFNγ	BV711	B27	BD Biosciences	564039
TNFα	BV421	MAb11	BD Biosciences	562783
IL2	PE	N7.48	Miltenyi	130-091-646
IL4	PERCPCY5.5	MP4-25D2	Biolegend	500822
FOXP3	AF700	PCH101	Thermo Fisher	56-4776-41
CXCR5	PE-Vio770	REA103	Miltenyi	130-117-358
PD1	BV786	EH12.1	BD Biosciences	563789
IL17	BV650	N49-653	BD Biosciences	563746
CCR7	BV711	3D12	BD Biosciences	563712
CD45RA	Pe-Texas-Red	MEM-56	Invitrogen	MHCD45RA17

**Other reagents**		
**Product**	**Supplier**	**Catalogous #**
Brilliant Violet Stain Buffer Plus	BD Biosciences	566385
FOXP3 buffer kit	Thermo fisher	00-5521-00
Zombie-red	Biolegend	423110

Supplementary Table 7. List of peptide-HLA tetramers used for flow cytometry

**Tetramers**		
**Peptide sequence (aa)**	**HLA allele**	**Supplier**
LTDEMIAQY	A*01:01	LUMC
FLPFFSNV	A*02:01	LUMC
RLNEVAKNL	A*02:01	LUMC
RLQSLQTYV	A*02:01	LUMC
VLNDILSRL	A*02:01	LUMC
YLQPRTFLL	A*02:01	LUMC
GTHWFVTQR	A*03:01/A*11:01	LUMC
KCYGVSPTK	A*03:01/A*11:01	LUMC
IYKTPPIKDF	A*24:02	LUMC
QYIKWPWYI	A*24:02	LUMC
RFDNPVLPF	A*24:02	LUMC
TQDLFLPFF	A*24:02	LUMC
TYVPAQEKNFT	A*24:02	LUMC
LPQGFSAL	B*07:02	LUMC
MIAQYTSAL	B*07:02	LUMC
SPRRARSVA	B*07:02	LUMC
CVADYSVLY	B*15:01	LUMC
LVKNKCVNF	B*15:01	LUMC
VASQSIIAY	B*15:01	LUMC
IYKTPPIKDF	B*35:01	LUMC
QPTESIVRF	B*35:01	LUMC

Supplementary Figure 3. Flow cytometry gating example for peptide stimulation assay and tetramer staining.

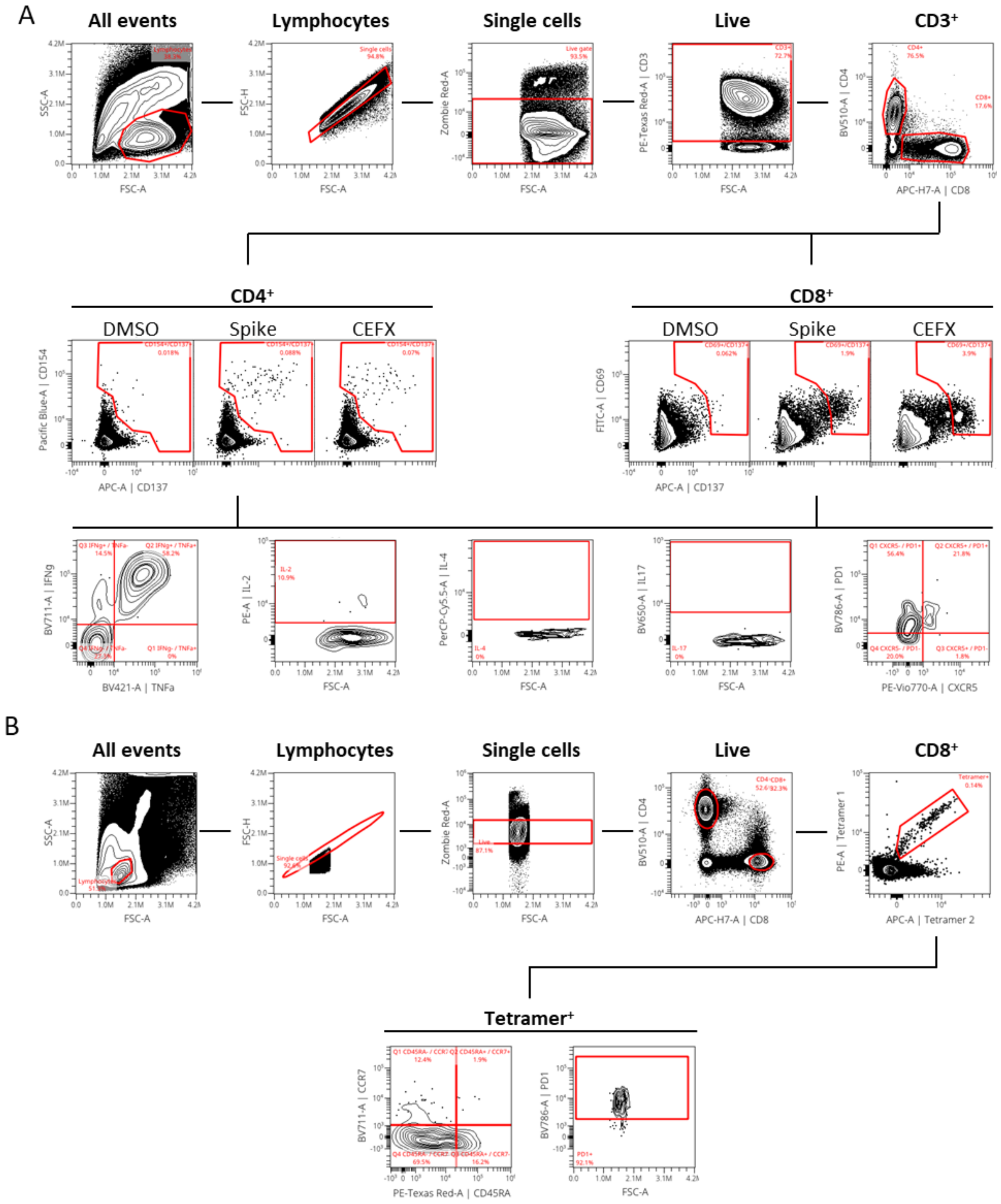

A. Representative example of flow cytometry gating strategy for peptide-reactive CD4^{+} and CD8^{+} T-cells. All events were gated on lymphocytes, single cells, viable cells, CD3^{+} positive and subsequently either CD4^{+} or CD8^{+} positive. For CD4^{+} T-cells, activated cells were gated on CD137^{+} and/or CD154^{+} positive whilst for CD8^{+} T-cells CD137^{+} and/or CD69^{+} positive cells were gated. DMSO functioned as a negative control and CEFX functioned as a positive control. From the activated cell gate onwards, cytokine production and marker expression was calculated. **B.** Representative example of flow cytometry gating strategy for spike-tetramer positive CD8^{+} T-cells. All events were gated on lymphocytes, single cells, viable cells, CD8^{+} positive and tetramer positive. Tetramer positive events were subsequently gated on CCR7, CD45RA or PD-1.

Supplement G: Supplementary Table 8. Characteristics of participants at inclusion

	**Overall (n=150)**	**20µg ID-SN (n=50)**	**20µg ID-BM (n=50)**	**100µg IM (n=50)**
Female, n (%)	63 (42)	17 (34)	24 (48)	22 (44)
Age, years Median (IQR)	22 (20-25)	21 (20-23)	22 (20-25)	23 (20-26)
BMI, kg/m^2 Median (IQR)	23.8 (21.0-26.9)	24.3 (20.9-28.6)	23.9 (21.0-27.2)	22.6 (21.0-26.4)

n = number of participants; ID=intradermal; IM= intramuscular; SN= standard needle; BM=Bella-mu® needle

Supplement H: Antibody response.

Supplementary Figure 4. Geometric mean concentrations and geometric mean fold rise of anti-RBD IgG in BAU/mL (95% CI).

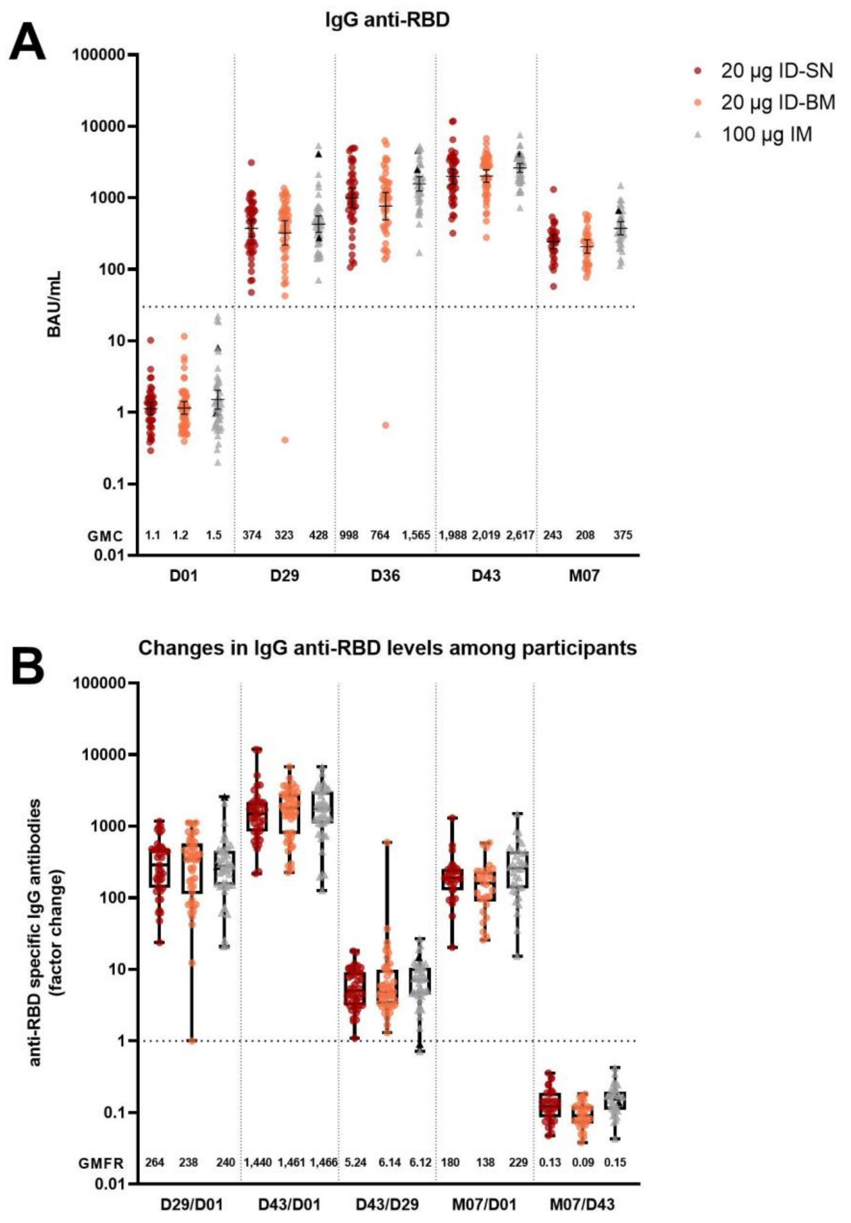

A. SARS-CoV-2 RBD-specific IgG antibody concentrations by bead-based immunoassay (MIA) in binding antibody units per mL in the three groups at each timepoint. Horizontal dotted lines represent the cut-off for seropositivity (= 30 BAU/mL). Horizontal lines represent the geometric mean + 95% CI of the geometric mean.
B. Per-participant factor changes for anti-RBD-specific binding antibodies, calculated by dividing two responses. The dashed line indicates a factor change of 1 (no increase or decrease).
For the calculation of the GMFR D29/D01, D43/D01 and M07/D01, any antibody concentration for RBD at day 1 reported below 1 was set to 1. For the calculation of the GMFR D43/D29, the antibody concentration for RBD for the non-responder in the ID-BM group at day 29 was set to 1.
Each symbol represents a sample from an individual participant. Black symbols in the IM group represent the two participants with SARS-CoV-2-spike-specific B-cells at baseline but no measurable anti-S or anti-N.
ID= intradermal; IM=intramuscular; BM= Bella-mu® needle; SN= standard needle.

Supplementary Table 9. Antibody concentrations reported in GMCs (IgG and neutralization) and GMTs (neutralization).

		20µg ID-SN	n	20µg ID-BM	n	100µg IM	n
**Day 01**	Anti-S1 IgG, BAU/mL (95% CI)	0.4 (0.3-0.5)	45	0.4 (0.3-0.6)	49	0.4 (0.3-0.6)	47
	Anti-RBD IgG, BAU/mL (95% CI)	1.1 (0.9-1.4)		1.2 (0.9-1.4)		1.5 (1.1-2.0)	
	Neutralization, ND_{50} (95% CI)	34.4 (31.0-38.1)		33.6 (31.3-36.)		34.1 (31.7-36.6)	
	Neutralization, IU/mL (95% CI)	8.7 (7.7-9.8)		8.3 (7.7-8.9)		8.4 (7.8-9.0)	
**Day 29**	Anti-S1 IgG, BAU/mL (95% CI)	591 (460-759)	43	537 (358-804)	46	692 (544-882)	45
	Anti-RBD IgG, BAU/mL (95% CI)	374 (85-491)		323 (218-479)		428 (329-556)	
	Neutralization, ND_{50} (95% CI)	1,487 (1,183-1,869)		1,488 (1,133-1,953)		1,871 (1,386-2,526)	
	Neutralization, IU/mL (95% CI)	366 (291-460)		366 (279-481)		460 (341-622)	
**Day 36**	Anti-S1 IgG, BAU/mL (95% CI)	1,539 (453-743)	42	1,251 (805-1,945)	44	2,461 (2,013-3,009)	42
	Anti-RBD IgG, BAU/mL (95% CI)	998 (723-1,379)		764 (491-1,190)		1,565 (1,247-1,965)	
	Neutralization, ND_{50} (95% CI)	3,704 (2,702-5,079)		3,455 (2,616-4,562)		5,607 (4,350-7,229)	41
	Neutralization, IU/mL (95% CI)	911 (665-1,250)		850 (644-1,123)		1,381 (1,072-1,782)	
**Day 43**	Anti-S1 IgG, BAU/mL (95% CI)	2,626 (2,076-3,322)	43	2,722 (2,248-3,296)	46	3,674 (3,202-4,215)	45
	Anti-RBD IgG, BAU/mL (95% CI)	1,988 (1,562-2,531)		2,019 (1,648-2,472)		2,617 (2,273-3,014)	
	Neutralization, ND_{50} (95% CI)	4,807 (3,673-6,291)		5,263 (4,144-6,686)		7,269 (6,046-8,739)	
	Neutralization, IU/mL (95% CI)	1,263 (951-1,676)		1,295 (1,020-1,645)		1,789 (1,488-2,150)	
**Month 07**	Anti-S1 IgG, BAU/mL (95% CI)	387 (303-493)	31	339 (268-428)	29	633 (508-790)	34
	Anti-RBD IgG, BAU/mL (95% CI)	243 (195-302)		208 (168-259)		375 (303-463.5)	
	Neutralization, ND_{50} (95% CI)	1,099 (850-1,420)	30	996 (727-1,365)	28	1,760 (1,331-2,327)	33
	Neutralization, IU/mL (95% CI)	270 (209-349)		271 (205-359)		433 (328-573)	

Conversion factor from neutralization (ND_{50}) to neutralization (IU/mL) is 4.06
IU/mL= International units per millimeter; BAU = Binding Antibody Units; CI = Confidence Intervals; n = number of participants; ID=intradermal; IM= intramuscular; SN= standard needle; BM=Bella-mu® needle; ND= neutralization dilution of 50%; RBD = receptor binding domain.

Supplementary Table 10. Fold change increase of GMC (IgG) and GMTs (neutralization), reported in GMFR.

		20µg ID-SN	n	20µg ID-BM	n	100µg IM	n
**D29/D01**	Anti-S1 IgG, GMFR (95% CI)	548 (424-707)	41	497 (340-729)	46	612 (488-767)	45
	Anti-RBD IgG, GMFR (95% CI)	264 (197-353)		238 (161-351)		240 (176-326)	
	Neutralization, GMFR (95% CI)	41.0 (33.3-50.4)		44.1 (33.8-57.6)		54.8 (41.8-71.8)	
**D43/D01**	Anti-S1 IgG, GMFR (95% CI)	2480 (1929-3187)	40	2480 (2009-3061)	46	3247 (2732-3860)	45
	Anti-RBD IgG, GMFR (95% CI)	1440 (1100-1885)		1461 (1139-1873)		1466 (1125-1910)	
	Neutralization, GMFR (95% CI)	144 (107-195)	41	156 (122-200)		213 (181-250)	
**D43/D29**	Anti-S1 IgG, GMFR (95% CI)	4.4 (3.6-5.3)	42	5.0 (3.7-6.8)	46	5.3 (4.3-6.6)	45
	Anti-RBD IgG, GMFR (95% CI)	5.24 (4.29-6.40)		6.14 (4.57-8.24)		6.12 (4.88-7.67)	
	Neutralization, GMFR (95% CI)	3.4 (2.6-4.5)	43	3.5 (2.7-4.6)		3.8 (3.0-4.9)	44
**M07/D01**	Anti-S1 IgG, GMFR (95% CI)	369 (285-479)	28	310 (290-399)	29	561 (434-724)	34
	Anti-RBD IgG, GMFR (95% CI)	180 (135-241)		138 (100-188)		229 (165-317)	
	Neutralization, GMFR (95% CI)	30.7 (22.1-42.6)		32.5 (24.3-43.5)	28	51.6 (38.8-68.7)	33
**M07/D43**	Anti-S1 IgG, GMFR (95% CI)	0.16 (0.13-0.19)	31	0.11 (0.1-0.13)	29	0.18 (0.15-0.21)	34
	Anti-RBD IgG, GMFR (95% CI)	0.13 (0.11-0.15)		0.09 (0.08-0.11)		0.15 (0.13-0.17)	
	Neutralization, GMFR (95% CI)	0.22 (0.16-0.30)	30	0.17 (0.13-0.23)	27	0.26 (0.20-0.33)	33

GMFR is calculated as the mean of the difference of logarithmically transformed assay results (late time point-earlier time point) and transformed back to the original scale. For the calculation of a more accurate GMFR, any antibody concentration for S1 and RBD at day 1 and 29 reported below 1 was set to 1. Results of the neutralization concentrations at D01 below the LLoD were arbitrarily set to LLoD/2.

GMFR= geometric mean fold rise; CI = Confidence Intervals; n = number of participants; ID=intradermal; IM= intramuscular; SN= standard needle; BM=Bella-mu® needle; ND= neutralization dilution of 50%; RBD = receptor binding domain.

Supplement I: SARS-CoV-2 specific B-cell response

To investigate the effect of ID-SN technique versus IM delivery of the mRNA-1273 SARS-CoV-2 vaccine on the B-cell compartment, the frequencies of SARS-CoV-2-specific B-cells after ID and IM delivery were compared using SARS-CoV-2-spike-fluorochrome-labbelled tetramers (Supplementary Figure 2). SARS-CoV-2-spike-specific B-cells were analyzed from 20 IM vaccinated and 20 ID vaccinated participants, balanced for age and gender. Two participants were found to have SARS-CoV-2-specific B-cells at day 1, followed by very high frequencies after vaccination, indicative of a prior infection (Supplementary Figure 5). These were shown as black datapoints in all figures and were excluded in the per-protocol analysis, but included in the intention-to-treat analysis. No differences were seen between these analyses.

To investigate the phenotype of SARS-CoV-2-specific B-cells, we first compared the frequencies of unswitched (IgD, IgM and IgMD) and switched (IgG and IgA) SARS-CoV-2-specific B cells (Supplementary Figure 7). Because the majority of SARS-CoV-2-specific B-cells class-switched to IgG at D43, we further focused on this memory IgG-compartment. To investigate the IgG+ SARS-CoV-2-specific B-cells phenotypical properties (e.g. migratory capacity (CXCR3), proliferating (Ki67+), activation status (CD95), enhancement of antibody production (CD21) and memory subsets, like CD45RB+ B-cells and CD11c+CD19^{high} cells[1]), a panel of 24 antibodies for phenotyping was used (Supplementary Table 4). In total, 43.532 SARS-CoV-2-specific B-cells were included from all samples. To visualize how the phenotype of SARS-CoV-2-specific B-cells changed over time, a Uniform Manifold Approximation and Projection (UMAP) dimensionality reduction was performed and the SARS-CoV-2-specific B-cells were visualized per time point in black (Supplementary Figure 6A). Migrating (CXCR3+), activated (CD95+) and memory (CD11c+CD19^{high}) SARS-CoV-2-specific IgG+ B-cells were most prevalent at day 43, while proliferating (Ki67+) were most prevalent at day 29 (Supplementary Figure 6B**,** Supplementary Figure 9). At 7 months, most SARS-CoV-2-spike-specific B-cells showed a resting phenotype. ID delivery of mRNA-1273 resulted in similar frequencies of IgG+ SARS-CoV-2 specific B-cell phenotypes as IM delivery.

Supplementary Figure 5. Participants with SARS-CoV-2-spike-specific B-cells prior to vaccination.

A

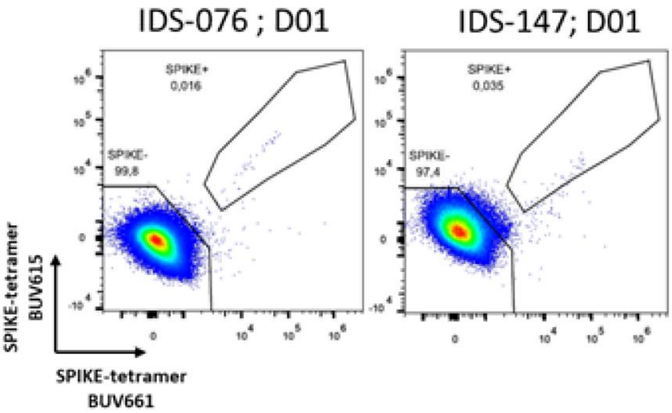

B

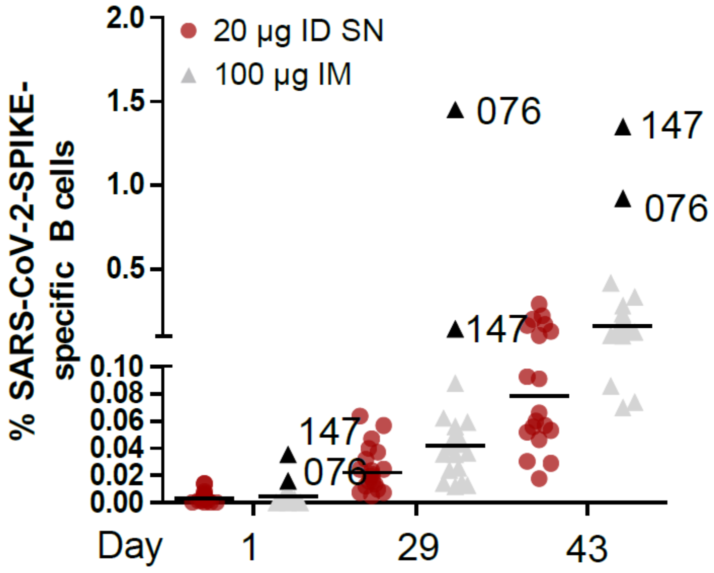

Shown are the results of two participants that contained SARS-CoV-2-specific B-cells prior to vaccination (Day 1). These participants are not included in the per-protocol analysis. Including these participants in an intention-to-treat analysis did not change the outcome.

Supplementary Figure 6. B-cell compartment and the immunogenicity of intradermal and intramuscular delivery of mRNA SARS-CoV-2 vaccine.

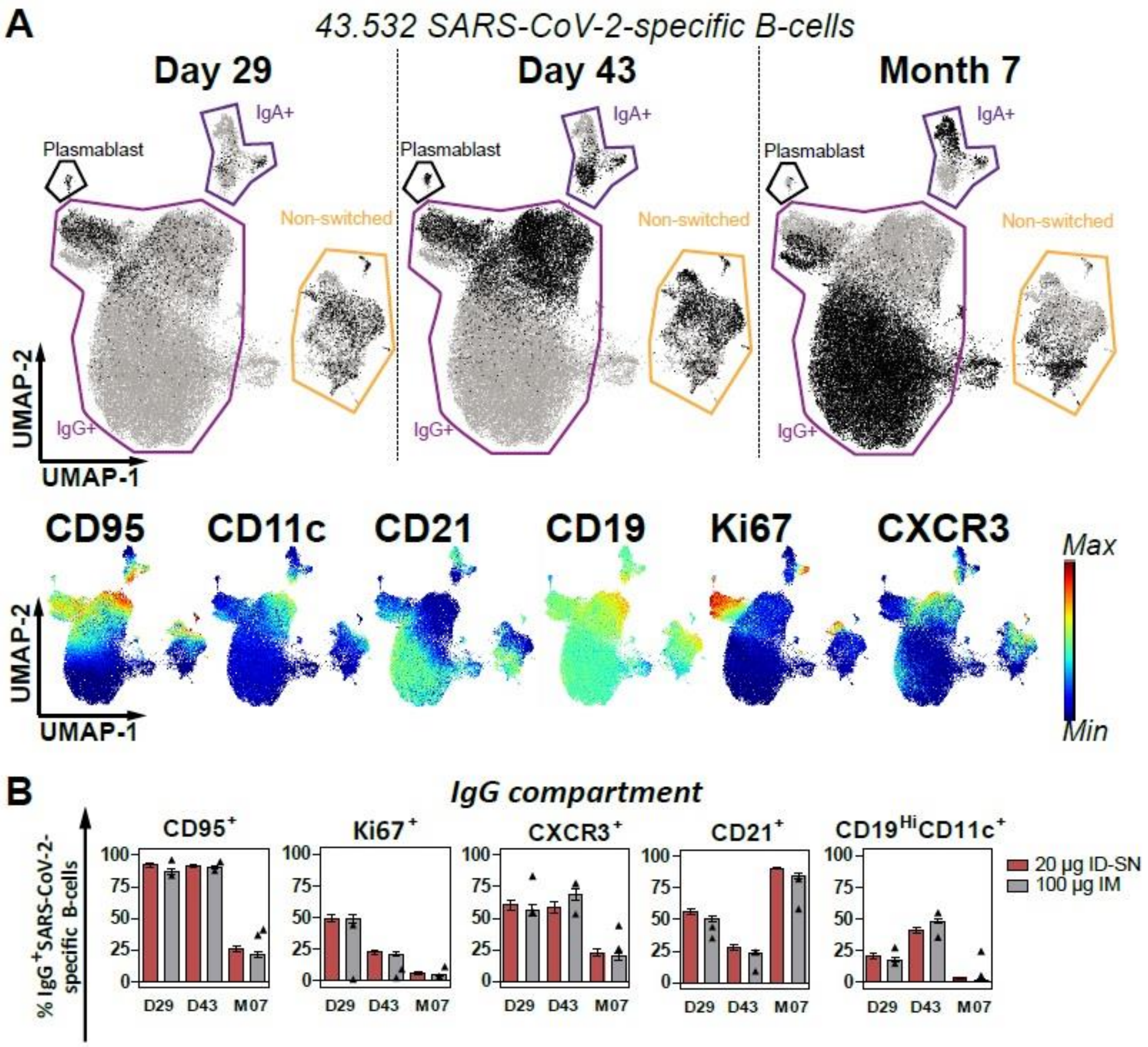

In total, 40 individuals were selected to investigate the B-cell response against the intramuscular (IM; 100µg) or intradermal (ID; 20µg) standard technique delivery of SARS-CoV-2 vaccine. 12 individuals withdrew from the study and two individuals (IM and ID group) were excluded at M07 and are not shown due to a recent breakthrough infection (PCR+). Vaccines were administered directly after sample collection at D01 and D29 (booster). The black data points represent the two individuals with the presence of SARS-CoV-2-specific B-cells at D01 prior to vaccination, which were excluded in the per-protocol analysis. Including these participants in the intention-to-treat analysis did not change the outcome.

A. Uniform Manifold Approximation and Projection (UMAP) for all 43.532 SARS-CoV-2-spike-specific B-cells (grey) to cluster cells based on 24 different markers. SARS-CoV-2-spike-specific B-cells are overlaid based on timepoint (black) on top of all cells (grey). Normalized expression of selected markers is shown below the overlaid UMAP. Expression plots for all markers can be found in Supplementary Figure 9. **B.** Specific markers and B-cell subsets are shown within the IgG+ B-cell compartment for each vaccine delivery (IM and ID) at each timepoint. Mean and standard error of mean are shown.

Statistical analyzes, Mann-Whitney U tests are performed to compare the vaccine deliveries (ID and IM) for each timepoint. *= P<0.05, **=P<0.01.

D=day; M=month

Supplementary Figure 7. Gating strategy of class-switching of SARS-CoV-2-specific B-cells.

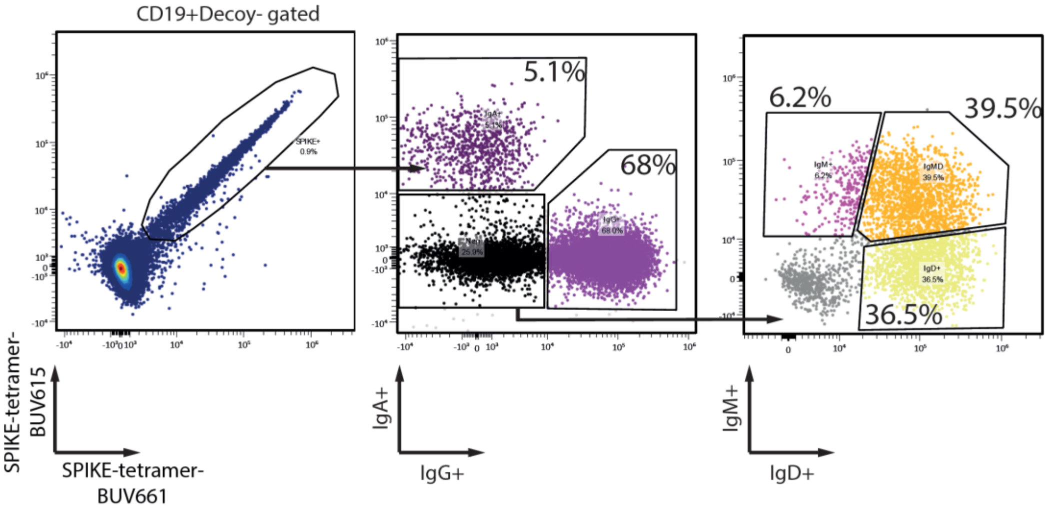

Isotype switching of B-cells was investigated by gating on IgD, IgM, IgMD, IgG and IgA expressing B-cells. Shown are plots of all samples (D29, D43, M07) that were concatenated. In total, 43.532 SARS-CoV-2-specific B-cells are shown.
D=day; M=month

Supplementary Figure 8. Class-switching of participants with SARS-CoV-2-spike-specific B-cells prior to vaccination.

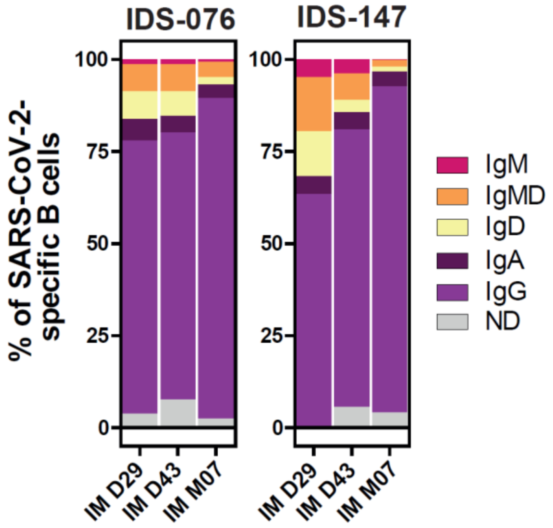

Isotype usage of SARS-CoV-2-spike-specific B-cells are shown as stacked bars at each timepoint for 2 participants that contained SARS-CoV-2-specific B-cells prior to vaccination (D01).
D=day; M=month

Supplementary Figure 9. Uniform Manifold Approximation and Projection (UMAP) of all SARS-CoV-2-specific B-cells.

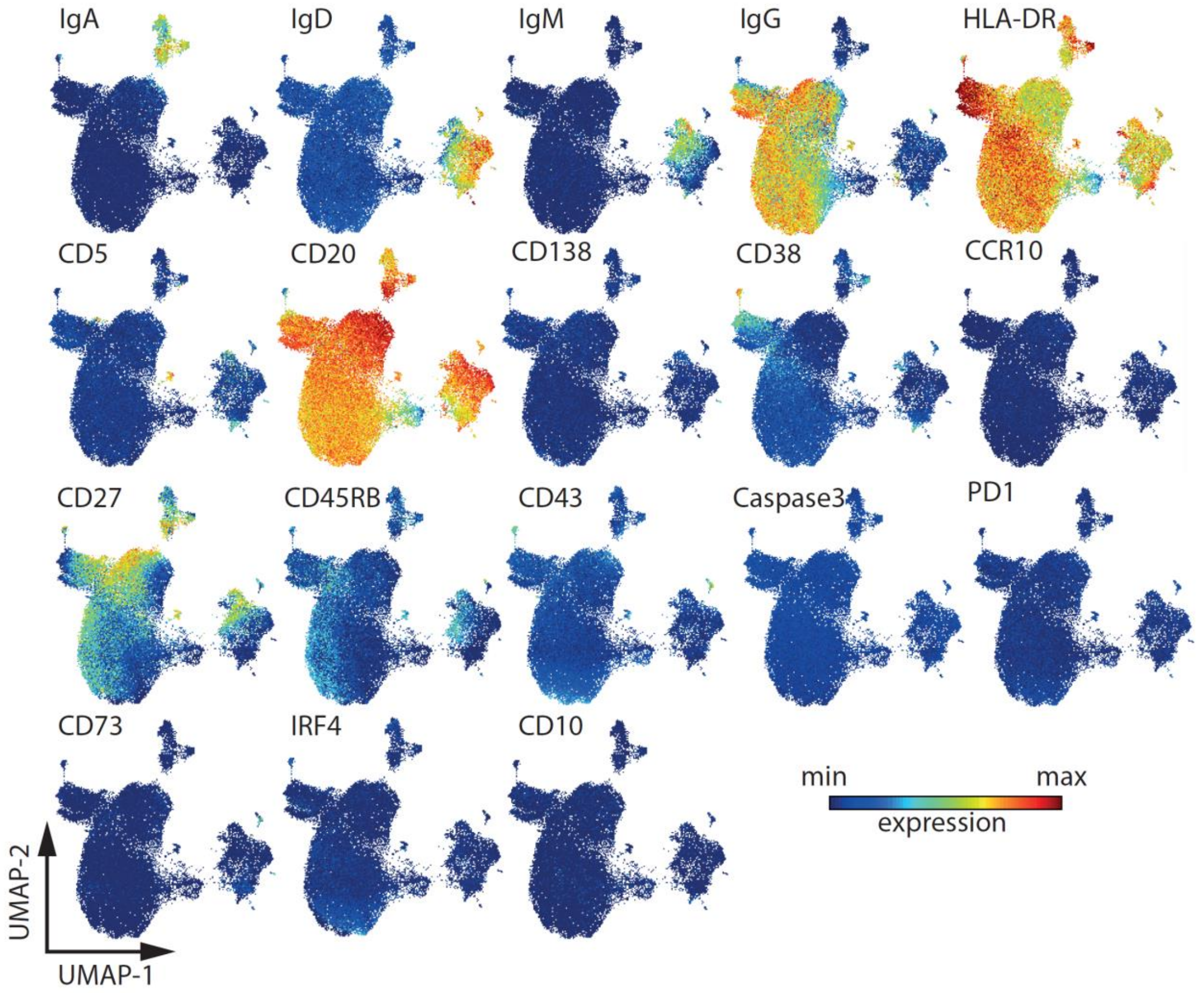

UMAPs of 43.532 SARS-CoV-2-specific B-cells are shown and overlaid with the normalized expression of markers that were used to define the phenotype of SARS-CoV-2-specific B-cells. The top row shows that the clustering/separation was largely due to the expression of different isotypes.

Supplement J: SARS-CoV-2 specific T-cell responses

Since the frequency of spike-specific T-cell responses was similar between the ID-SN and IM group, the function of the SARS-CoV-2-reactive T-cells was further evaluated by measuring the percentages of SARS-CoV-2-spike-specific Th1 (IFN-γ, TNF-α or IL-2), Th2 (IL-4), Th17 (IL-17), Tfh ($PD1^+CXCR5^+$), and Treg (IFN-γ^-TNF-α^- $FOXP3^+$). The profile of cytokine production was different between the $CD4^+$ and $CD8^+$ T-cells (Supplementary Figure 10A and 10B). IL-2 responses were not seen in $CD8^+$ T-cells. In both groups, the spike-specific $CD4^+$ T-cells primarily exhibited a Th1 cell response, as they mostly produced IFN-γ, TNF-α and IL-2. In addition, increased expression of PD-1 was observed. Slight reductions were observed in TNF-α and IL-2 expression in the IM group compared to the ID-SN group, with only the latter being significant (Supplementary Figure 10A). The frequency of SARS-CoV-2-reactive $CD4^+$ and $CD8^+$ T-cells that produced IL-2, IFN-γ and TNF-α was similar between the two groups. The SARS-CoV-2-specific $CD8^+$ T-cells induced by ID and IM produced both IFN-γ and TNF-α in most individuals and showed increased PD-1 expression (Supplementary Figure 10B). None of these parameters were significantly different between the two cohorts.

Finally, the differentiation state of vaccine-induced $CD8^+$ T-cells was studied, and showed that in both IM and ID-SN groups, SARS-CoV-2-specific $CD8^+$ T-cells, measured by pHLA tetramer binding, mainly appear to have an effector memory (EM) phenotype (Supplementary Figure 10C).

Supplementary Figure 10. mRNA-1273 induced SARS-CoV-2-specific T-cell responses

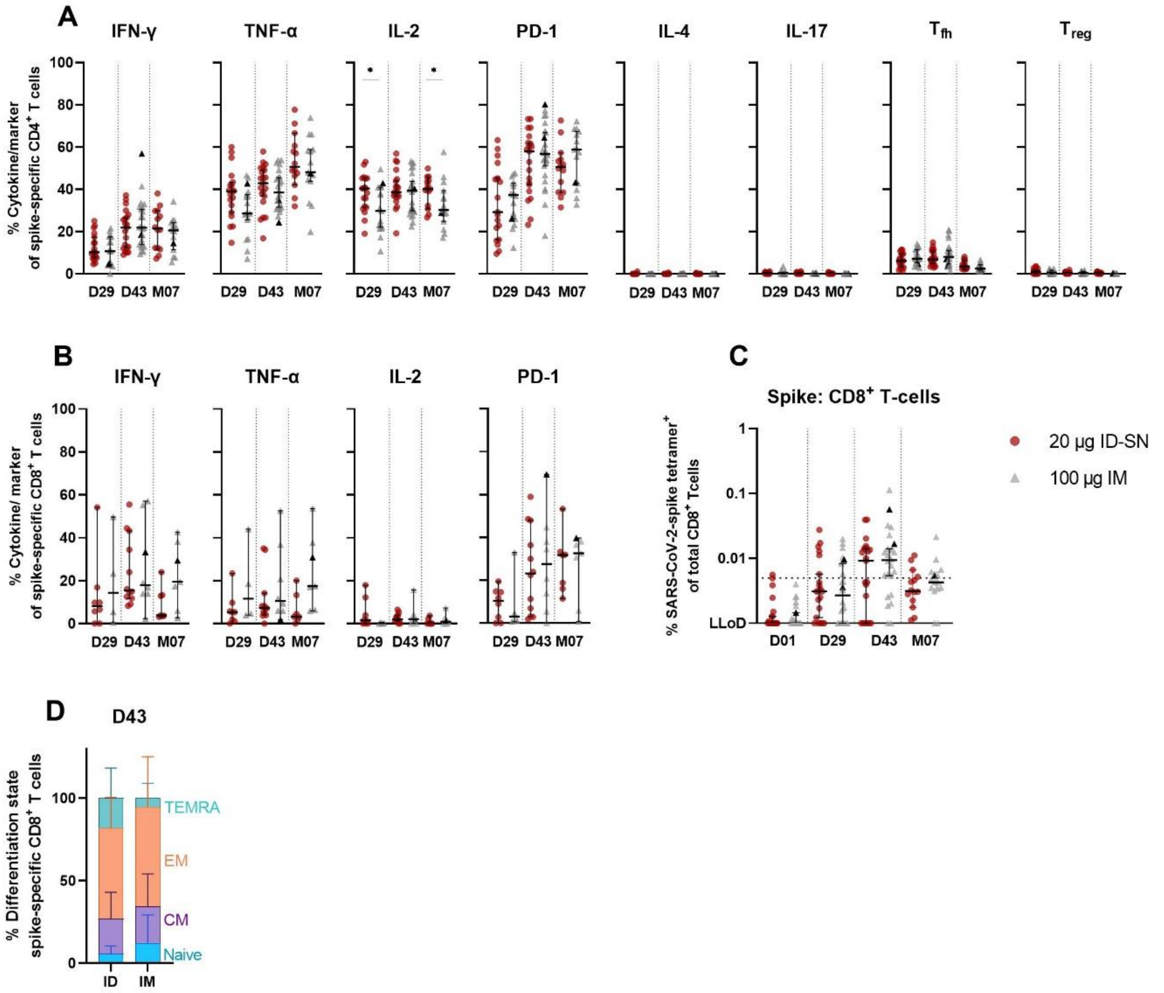

A. Frequency of spike-specific $CD4^+$ T-cells that are positive for depicted cytokines or markers. T follicular helper cells (T_{fh}) were defined as $CXCR5^+PD\text{-}1^+$, PD-1 as $CXCR5^-PD\text{-}1^+$ and regulatory T-cells (T_{reg}) as $FOXP3^+IFN\text{-}\gamma^-TNF\text{-}\alpha^-IL\text{-}2^-$. **B.** Frequency of spike-specific $CD8^+$ T-cells that are positive for depicted cytokines or markers. **C**. Frequency of spike-specific $CD8^+$ T-cells in time. Spike-specific $CD8^+$ T-cells were measured by peptide-HLA tetramer technology. Dotted line represents threshold for a response. **D.** Frequency of spike-specific $CD8^+$ T-cells that are naïve (blue; $CCR7^+CD45RA^+$), central memory (CM; purple;$CCR7^+CD45RA^-$), effector memory (EM; orange;$CCR7^-CD45RA^+$) or terminal effector memory (TEMRA; green;$CCR7^-CD45RA^+$).

Each point represents a single subject. Black symbols in the IM group represent the two participants with suspected previous SARS-CoV-2 infection, based on SARS-CoV-2-spike-specific B-cells prior to vaccination. Mann-Whitney U tests were performed for statistical analysis. *p*-values are categorized in the figures as: **p*<0.05; ***p*<0.01 or ****p*<0.001. Categorized *p*-value was only shown if significant.

Horizontal bold black line represents the median with 95% CI. The dotted line indicates limit of quantification (LOQ).

ID= intradermal; IM=intramuscular; SN= standard needle; LLoD= lower limit of detection; CI= confidence interval; D=day; M=month

Supplement K: Adverse events

Supplementary Table 11. Number of participants who used antipyretics for related adverse events.

	20µg ID-SN	20µg BM	100µg IM
**Vaccination 1**	4/50 (8.0%)	7/50 (14.0%)	4/50 (8.0%)
**Vaccination 2**	11/45 (24.4%)	5/48 (10.4%)	21/48 (43.8%)

ID=intradermal; IM= intramuscular; SN= standard needle; BM=Bella-mu® needle

Supplementary Table 12. Local and systemic adverse events, related to the vaccination.

**Vaccination 1**		**20µg ID-SN (n=50)**	**20µg ID-BM (n=50)**	**100µg IM (n=50)**
Any local symptoms, n (%)	Mild	47 (94)	44 (88)	44 (88)
	Moderate	14 (28)	14 (28)	28 (56)
	Severe	1 (2)	0 (0)	3 (6)
Any systemic symptoms, n (%)	Mild	30 (60)	29 (58)	35 (70)
	Moderate	15 (30)	11 (22)	14 (28)
	severe	1 (2)	1 (2)	3 (6)
**Vaccination 2**		**20µg ID-SN (n=50)**	**20µg ID-BM (n=50)**	**100µg IM (n=50)**
Any local symptoms, n (%)	Mild	41 (91)	39 (81)	33 (69)
	Moderate	21 (47)	25 (52)	38 (79)
	Severe	1 (2)	2 (4)	8 (17)
Any systemic symptoms, n (%)	Mild	31 (69)	31 (65)	41 (85)
	Moderate	15 (33)	5 (10)	26 (54)
	severe	3 (7)	0 (0)	9 (19)

Any local or systemic adverse events are given when a participant experienced one or more local or systemic (mild, moderate or severe) adverse event. All adverse events possibly, probably or definitely related to the vaccination in the following 28 days after the first and second vaccination are reported.

n = number of participants; ID=intradermal; IM= intramuscular; SN= standard needle; BM=Bella-mu® needle

Supplementary Table 13. Adverse events related to vaccine administration after vaccination one and two.

		Number of reported adverse events after vaccination one in percentages								
		**20µg ID-SN (n=50)**			**20µg ID-BM (n=50)**			**100µg IM (n=50)**		
		**Mild**	**Moderate**	**Severe**	**Mild**	**Moderate**	**Severe**	**Mild**	**Moderate**	**Severe**
**Local**	Hyperpigmentation	2	0	0	4	0	0	0	2	0
	Axillar lymphadenopathy	6	0	0	20	0	0	10	2	0
	Swelling	10	8	8	20	8	8	10	8	2
	Itch	44	4	0	40	0	0	14	0	0
	Local muscle stiffness	36	2	0	42	4	0	54	40	2
	Erythema	40	14	0	52	14	0	12	8	2
	Pain at injection site	70	10	2	64	12	0	74	18	2
**Systemic**	Fever	0	0	0	0	0	0	2	0	0
	Dizziness	4	2	0	0	0	0	2	0	0
	Nausea & vomiting	2	4	0	4	0	0	6	0	0
	Diarrhoea	0	2	0	6	0	0	8	0	0
	Arthralgia	0	0	0	6	0	0	6	2	2
	Myalgia	8	4	0	12	0	0	10	10	0
	Chills	12	0	0	2	2	0	12	4	0
	Headache	28	8	0	36	12	0	40	6	2
	Fatigue & malaise	34	18	2	40	14	2	38	22	2
		**Number of reported adverse events after vaccination two in percentages**								
		**20µg ID-SN (n=45)**			**20µg ID-BM (n=48)**			**100µg IM (n=48)**		
		**Mild**	**Moderate**	**Severe**	**Mild**	**Moderate**	**Severe**	**Mild**	**Moderate**	**Severe**
**Local**	Hyperpigmentation	0	0	0	4	0	0	0	0	0
	Axillar lymphadenopathy	20	2	0	4	2	0	17	4	0
	Swelling	24	11	0	29	8	2	2	10	2
	Itch	31	2	0	29	0	0	4	0	0
	Local muscle stiffness	29	9	0	29	6	0	27	65	8
	Erythema	33	33	2	33	40	4	4	4	4
	Pain at injection site	56	22	0	54	21	0	40	44	13
**Systemic**	Fever	9	0	0	2	0	0	15	6	6
	Dizziness	2	0	0	4	0	0	2	0	0
	Nausea & vomiting	7	0	0	2	0	0	8	4	0
	Diarrhoea	2	0	0	10	0	0	6	0	0
	Arthralgia	7	2	0	2	0	0	19	8	0
	Myalgia	13	4	0	10	0	0	23	21	0
	Chills	20	4	2	17	0	0	48	19	2
	Headache	31	22	2	35	2	0	38	23	8
	Fatigue & malaise	31	18	4	42	10	0	40	27	8

ID= intradermal; IM= intramuscular; SN= standard needle; BM= Bella-mu® needle

Supplementary Table 14. Numbers of participants with recurrence of erythema and swelling >2.5 cm after vaccination one and two.

	20µg ID-SN		20µg ID-BM		100µg IM	
	**Erythema**	**Swelling**	**Erythema**	**Swelling**	**Erythema**	**Swelling**
**Vaccination 1**	14/50 (28%)	6/50 (12%)	15/50 (30%)	11/50 (22%)	6/50 (12%)	4/50 (8%)
**Vaccination 2**	0/45	0/45	0/48	0/48	0/48	0/48

ID= intradermal; IM= intramuscular; SN= standard needle; BM= Bella-mu® needle

Supplementary Figure 11. Recurrence of erythema and swelling.

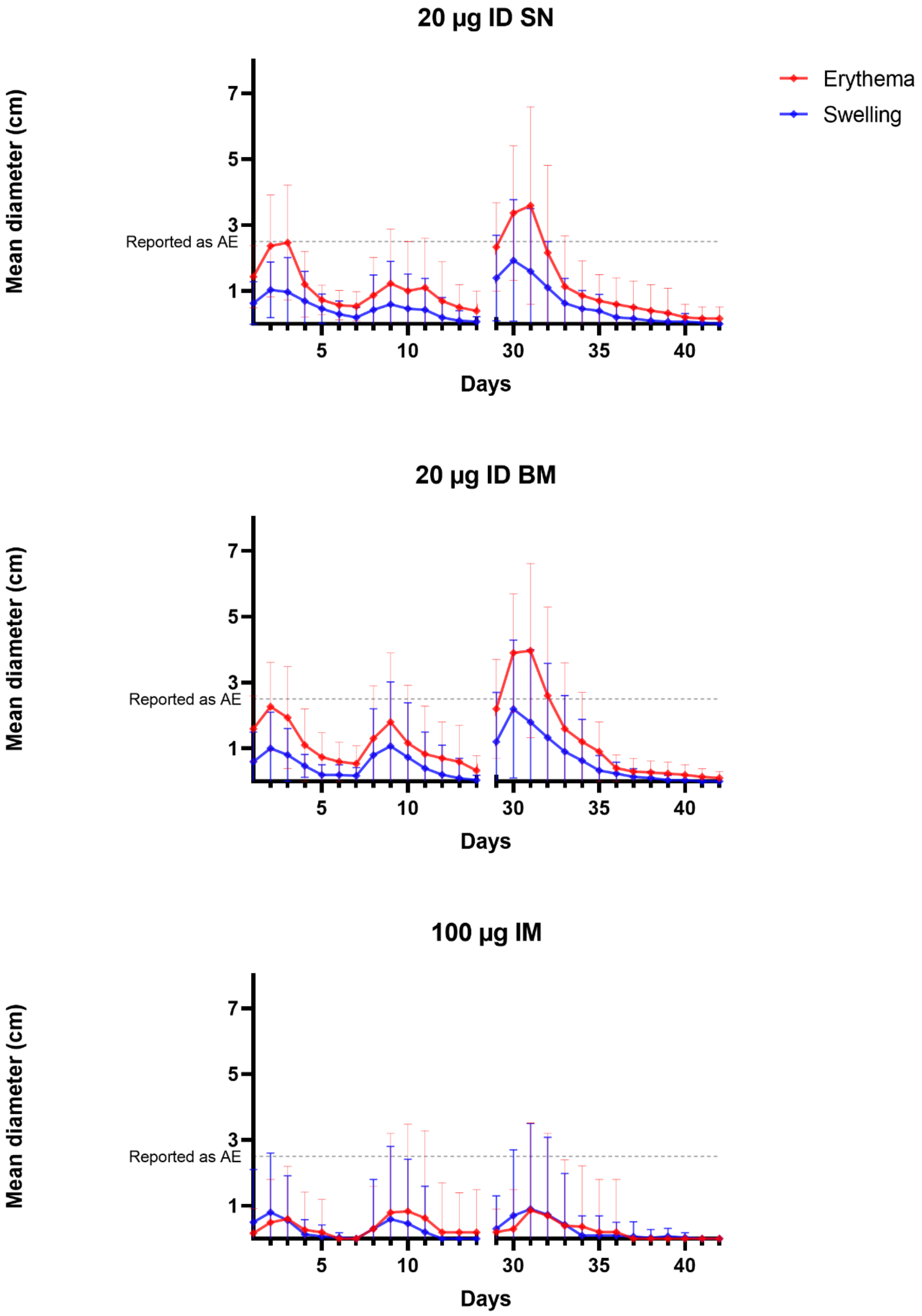

In some individuals, particularly in the ID group, a transient recurrence of erythema and swelling (sometimes referred as 'COVID-arm'[2-4]) occurred around day eight after resolution of initial local symptoms (see also Supplementary Table 14).

ID=intradermal; IM= intramuscular; SN= standard needle; BM= Bella-mu® needle.

Supplementary Table 15. Number of participants that reported a severe adverse event.

	20µg ID-SN	20µg ID-BM	100µg IM
**Local or systemic severe adverse event**			
Vaccination 1	2/50 (4%)	1/50 (2%)	5/50 (10%)
Vaccination 2	4/45 (8.9%)	2/48 (4.2%)	12/48 (25%)
**Local severe adverse event**			
Vaccination 1	1/50 (2%)	-	3/50 (6%)
Vaccination 2	1/45 (2.2%)	2/48 (4.2%)	8/48 (16,7%)
**Systemic severe adverse event**			
Vaccination 1	1/50 (2%)	1/50(2%)	3/50 (6%)
Vaccination 2	3/45 (6.7%)	-	9/48 (18,8%)

ID=intradermal; IM= intramuscular; SN= standard needle; BM=Bella-mu® needle

Supplementary Table 16. Numbers of severe adverse events reported by the participants.

	20µg ID-SN	20µg ID-BM	100µg IM	Duration, median (IQR)
Local muscle stiffness	-	-	5	4 (3-4)
Pain at injection site	1	-	7	4 (3-4)
Swelling	-	1	2	4 (3-6)
Erythema	1	2	3	5 (4-5)
Headache	1	-	5	3 (2-4)
Fever	-	-	3	1 (1-2)
Chills	1	-	1	3 (2-3)
Malaise	3	1	5	3 (3-4)
Arthralgia	-	-	1	2 (2-2)
**Total**	**7**	**4**	**32**	

ID=intradermal; IM= intramuscular; SN= standard needle; BM=Bella-mu® needle

Supplementary References

1. Golinski ML, Demeules M, Derambure C, et al. CD11c(+) B Cells Are Mainly Memory Cells, Precursors of Antibody Secreting Cells in Healthy Donors. Front Immunol. 2020;11:32.
2. Ramos CL, Kelso JM. "COVID Arm": Very delayed large injection site reactions to mRNA COVID-19 vaccines. J Allergy Clin Immunol Pract. 2021;9(6):2480-1.
3. Wei N, Fishman M, Wattenberg D, et al. "COVID arm": A reaction to the Moderna vaccine. JAAD Case Rep. 2021;10:92-5.
4. Blumenthal KG, Freeman EE, Saff RR, et al. Delayed Large Local Reactions to mRNA-1273 Vaccine against SARS-CoV-2. N Engl J Med. 2021;384(13):1273-7.
